# Uncovering hidden phylo- and ecogenomic diversity of the widespread methanotrophic genus *Methylobacter*

**DOI:** 10.1093/femsec/fiaf127

**Published:** 2025-12-13

**Authors:** Magdalena Wutkowska, Justus A Nweze, Vojtěch Tláskal, Julius E Nweze, Anne Daebeler

**Affiliations:** Institute of Soil Biology and Biogeochemistry, Biology Centre CAS, Na Sádkách 702/7, 370 05 České Budějovice, Czech Republic; Institute of Soil Biology and Biogeochemistry, Biology Centre CAS, Na Sádkách 702/7, 370 05 České Budějovice, Czech Republic; Department of Ecosystem Biology, Faculty of Science, University of South Bohemia, Branišovská 31a, 370 05 České Budějovice, Czech Republic; Department of Science Laboratory Technology, Faculty of Physical Sciences, University of Nigeria, Owerre Eze Rd, Ihe Nsukka, Nsukka 410105, Nigeria; Institute of Soil Biology and Biogeochemistry, Biology Centre CAS, Na Sádkách 702/7, 370 05 České Budějovice, Czech Republic; Institute of Soil Biology and Biogeochemistry, Biology Centre CAS, Na Sádkách 702/7, 370 05 České Budějovice, Czech Republic; Institute National de Recherche Scientifique, Centre Armand Frappier Santé et Biotechnologie, Laval, QC H7V 1B7, Canada; Institute of Soil Biology and Biogeochemistry, Biology Centre CAS, Na Sádkách 702/7, 370 05 České Budějovice, Czech Republic

**Keywords:** aerobic methanotrophy, gas vesicles, genome-resolved taxonomy, metabolic versatility, oxygen-depleted environment, soluble methane monooxygenase

## Abstract

The globally distributed genus *Methylobacter* plays a crucial role in mitigating methane emissions from diverse ecosystems, including freshwater and marine habitats, wetlands, soils, sediments, groundwater, and landfills. Despite their frequent presence and abundance in these systems, we still know little about the genomic adaptations that they exhibit. Here, we used a collection of 97 genomes and metagenome-assembled genomes to ecogenomically characterize the genus. Our analyses suggest that the genus *Methylobacter* may contain more species than previously thought, with >30 putative species clusters. Some species clusters shared >98.65% sequence identity of the full-length 16S rRNA gene, demonstrating the need for genome-resolved species delineation. The ecogenomic differences between *Methylobacter* spp. include various combinations of methane monooxygenases, multigene loci for alternative dissimilatory metabolisms related to hydrogen, sulfur cycling, and denitrification, as well as other lifestyle-associated functions. Additionally, we describe and tentatively name the two new *Methylobacter* species, which we recently cultured from sediment of a temperate eutrophic fishpond, as *Methylobacter methanoversatilis*, sp. nov. and *Methylobacter spei*, sp. nov. Overall, our study highlights previously unrecognized species diversity within the genus *Methylobacter*, their diverse metabolic potential, versatility, as well as the presence of distinct genomic adaptations for thriving in various environments.

## Teaser

The genus *Methylobacter* is far more diverse and ecologically versatile than previously recognized, comprising over 30 putative species with distinct genomic adaptations and metabolic traits, including two newly cultured species, underscoring its key role in global methane mitigation across diverse environments.

## Introduction

Methane (CH_4_) is a potent greenhouse gas with an 84-fold greater warming potential than carbon dioxide (CO_2_) over the first 20 years after release (Myhre et al. 2013). However, the overall radiative forcing caused by CH_4_ is likely heavily underestimated by ~25% (Etminan et al. 2016). The CH_4_ level in Earth’s atmosphere has been steadily increasing since the Industrial Revolution. In 2025, the global monthly mean abundance of atmospheric CH_4_ concentration surpassed 1.93 ppm (Lan et al. 2022; version 2025–09), nearly tripling the preindustrial 0.7 ppm (Sapart et al. 2012). The largest natural sources of CH_4_ are global freshwater systems, including wetlands, which contribute over 50% of total emissions (Jackson et al. 2020). Methanotrophs, microorganisms that can use CH_4_ as their only carbon and energy source, can consume >90% of *in situ*-produced CH_4_ before it reaches the atmosphere (King 1990, King et al. 1990, Oremland and Culbertson 1992, Michaud et al. 2017). However, it is uncertain whether the efficiency of CH_4_ removal can be sustained in the face of the rapid progression of climate change.

In many ecosystems that produce large amounts of CH_4_, gammaproteobacterial methanotrophs from the genus *Methylobacter* can dominate the active methanotrophic community (e.g. Nercessian et al. 2005, Tveit et al. 2013, Rissanen et al. 2018, Smith et al. 2018, Savvichev et al. 2021, Deng et al. 2024, Li et al. 2025, Wang et al. 2025). Members of the genus *Methylobacter* are widespread, having been identified in samples collected from all continents, predominantly in diverse freshwater, wetland, and soil habitats (Rodrigues et al. 2025). They are known to oxidize CH_4_ with the particulate CH_4_ monooxygenase (pMMO), assimilate carbon through the ribulose monophosphate pathway (RuMP), and use ubiquinone Q-8 as a major respiratory lipoquinone (Bowman et al. 1993, Bowman 2006). The majority of *Methylobacter* isolates grow relatively quickly and efficiently oxidize CH_4_, even at low temperatures (Tveit et al. 2023). In fully oxic conditions, CH_4_ oxidation rates can reach up to 0.60 μmol CH_4_ 10^8^ cells^−1^ h^−1^ for *M. luteus* (Tveit et al. 2023). The cultured species grow optimally at temperatures between 23°C and 30°C (Bowman et al. 1993, Hanson and Hanson 1996, Bowman 2014, Bodelier et al. 2019), with few psychrophilic strains (Omelchenko et al. 1993, Khanongnuch et al. 2022, Patil et al. 2024), widespread psychrotolerance (Wartiainen et al. 2006, Roldán and Menes 2023), and only some thermotolerant species with optima >35°C (Lidstrom 1988). *Methylobacter* spp., although geographically widely distributed, are predominantly found in nonacidic pH ranges (Seppey et al. 2023), with a few exceptions (Nguyen et al. 2018, Hogendoorn et al. 2021, Nweze et al. 2024).

The genus *Methylobacter* was initially named in 1970 (Whittenbury et al. 1970, 1970). However, the name was only officially (taxonomically) introduced two decades later, reclassifying known methanotrophs based on the DNA–DNA hybridization values and phospholipid fatty acid compositions (Bowman et al. 1993). A genome-based reclassification of methanotrophs, including the genus *Methylobacter*, corrected some misclassifications, which had previously contributed to a polyphyletic character of the genus, and identified 10 species-level clusters in the genus *Methylobacter* (Orata et al. 2018).

Despite their widespread occurrence across these diverse ecosystems and the abundance of genomic data available, *Methylobacter* spp. have not been systemically studied to uncover their putative genomic adaptations. In this study, we aim to close this knowledge gap, by using 97 genomes and MAGs classified as *Methylobacter*, retrieved in May 2024 from the NCBI Genome portal, to comprehensively characterize them regarding their potential metabolic capabilities in the context of phylogenomics. A special focus was directed toward the presence and genomic organization of different CH_4_ monooxygenase forms, dissimilatory metabolisms other than CH_4_ oxidation, as well as genomic adaptations to diverse environmental conditions, including O_2_-depleted habitats. Additionally, we characterize two recently obtained novel *Methylobacter* spp. (Wutkowska and Daebeler 2024), which we tentatively name *Methylobacter spei*, sp. nov., and *Methylobacter methanoversatilis*, sp. nov.

## Materials and methods

### *Methylobacter* genomes and MAGs

**Figure 1.**
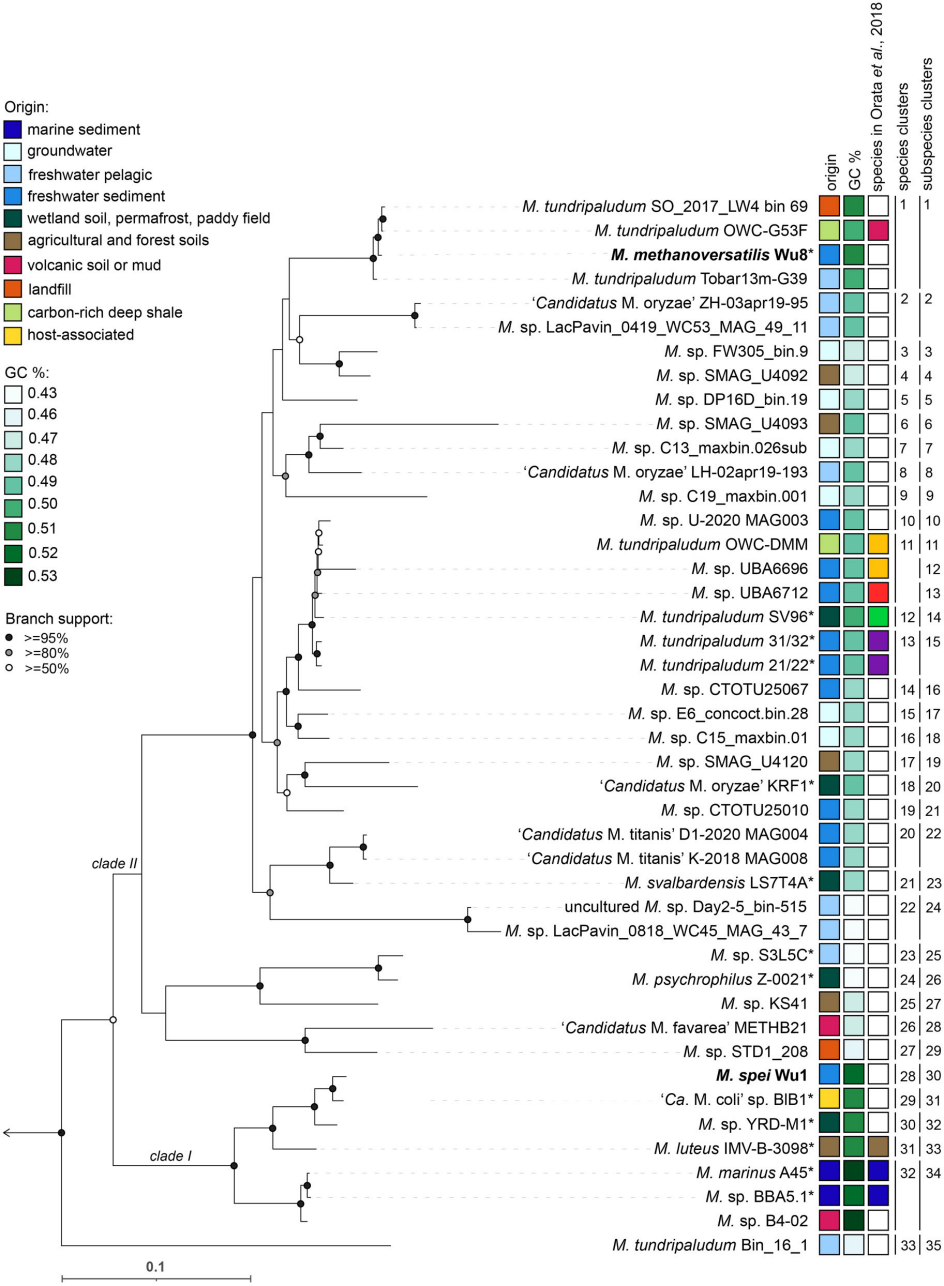
Phylogeny of the genus *Methylobacter* based on concatenated amino acid sequences of 71 single-copy genes from 44 high-quality nonredundant *Methylobacter* spp. genomes and MAGs. The name “*Methylobacter*” has been replaced by “*M*.”. Genomes from axenic cultures are indicated by asterisks (*), and genomes from our recently cultured strains are shown in bold. Colored squares indicate origin habitat type, GC content, and species included and delineated in Orata et al. (2018) (each color represents a distinct species). Numbers and vertical lines indicate species and subspecies inferred by the Type-Strain-Genome-Server. The genome of *Methylomicrobium lacus* LW14 (Kalyuzhnaya et al. 2015) was used as an outgroup and indicated by the arrow pointing outwards placed on the first node of the tree. Branch bootstrap support is indicated on the nodes as black (≥95%), gray (≥80%), or white (>50%) circles. The scale bar indicates 0.1 amino acid substitutions per site.

On 21 May 2024, we retrieved all 97 available genomes and MAGs classified as *Methylobacter* from the NCBI Genome database (Table S1, Fig. 1). Genomes of isolates included: *M. luteus* 98 or IMV-B-3098, previously *M. bovis* (Whittenbury et al. 1970, 1970)*; M. whittenburyi* (formerly *M. capsulatus* UCM-B-3033) (Whittenbury et al. 1970, Hamilton et al. 2015); *M. marinus* A45 (formerly *Methylomonas methanica* A4) (Lidstrom 1988, Flynn et al. 2016); *Methylobacter* sp. BBA5.1 (Smith et al. 1997, Flynn et al. 2016), *M. tundripaludum* SV96 (Wartiainen et al. 2006, Svenning et al. 2011); *M. psychrophilus* Z-0021 (Omelchenko et al. 1993, 1996, Rissanen et al. 2022); *Methylobacter* sp. BBA5.1 (Smith et al. 1997, Flynn et al. 2016); *Methylobacter* sp. 21/22 and 31/32 (Beck et al. 2013, Kalyuzhnaya et al. 2015, Oshkin et al. 2015); “*Ca*. M. oryzae” KRF1 (Khatri et al. 2020); “*Ca*. M. coli” BlB1 (Khatri et al. 2021); *Methylobacter* sp. YRD-M1 (Hao et al. 2022), *Methylobacter* sp. S3L5C (Khanongnuch et al. 2022), *Methylobacter* sp. Wu8 (Wutkowska and Daebeler 2024), and *M. svalbardiensis* LS7-T4A (Patil et al. 2024). A recent reclassification proposed *M. whittenbury* and *M. marinus* to be the heterotypic synonyms, as their genomes share a very high degree of similarity (89% dDDH and 99% ANI) (Orata et al. 2018), with *M. marinus* being the recommended valid name, therefore we did not include a *M. whittenbury* genome in our analyses.

*Methylobacter* spp. that have been sequenced from enrichment cultures belong to: *Methylobacter* sp. KS41 (Nguyen et al. 2018); “*Ca*. M. titanis” sp. nov. strains K-2018 and D1-2020, as well as *Methylobacter* sp. U-2020 MAG003 (Roldán and Menes 2023), and *Methylobacter* sp. strains Wu1 (Wutkowska and Daebeler 2024). The remaining MAGs originated from environmental samples (e.g. Parks et al. 2017, Woodcroft et al. 2018, Pedron et al. 2019, Zheng et al. 2020, Buck et al. 2021, Hogendoorn et al. 2021, Rissanen et al. 2021, Chiriac et al. 2022, Magnuson et al. 2022, Cabello‐Yeves et al. 2023, Grégoire et al. 2023, Jaffe et al. 2023, Ma et al. 2023, Ruff et al. 2023, Slobodkin et al. 2023, Rocha et al. 2024).

The gene coding sequences were predicted and translated to amino acid sequences using Prodigal v.2.6.3 (Hyatt et al. 2010). To assess similarity among the 97 genomes, we calculated average nucleotide identity inferred with *blastp* (ANIb) using pyANI (Pritchard et al. 2016) in-built in a pangenome workflow in anvio v.7.1 “Hope” (Eren et al. 2021) with third party software DIAMOND (Buchfink et al. 2021) and MUSCLE (Edgar 2004). To reduce redundancy in our dataset, genomes and MAGs with >99% ANIb similarity were grouped, and a representative genome for each group was chosen based on the highest completeness and lowest contamination calculated with CheckM2 v.1.0.1 (detailed summary available in Table S1) (Chklovski et al. 2023). Those genomes with >90% completeness and <5% contamination was categorized as “high-quality,” although they sometimes consist of many contigs. Subsequently, only representative high-quality genomes and MAGs (*n* = 44) were retained for all downstream analyses (Table S1, Fig. 1).

### Phylogenomic analysis, genus and species delineation

The phylogenomic analysis was performed using Anvi’o in the Anvi’o development environment (anvio-dev) (Eren et al. 2021). The FASTA files containing genome sequences were reformatted to meet Anvi’o’s contigs database requirements using the anvi-script-reformat-fasta command. For each reformatted FASTA file, an Anvi’o contigs database was generated using the anvi-gen-contigs-database command, which used Prodigal (v2.6.3) (Hyatt et al. 2010) to identify open reading frames in the DNA sequences and predict protein-coding genes. Conserved genes, including single-copy core genes (SCGs), were annotated and identified using hidden Markov models (HMMs) with the anvi-run-hmms command and the Bacteria_71 HMM profile. The sequences of the best hit for each SCG were extracted and concatenated into a single FASTA file using the anvi-get-sequences-for-hmm-hits command. The concatenated protein sequences were aligned using MAFFT (v7.520) (Katoh and Standley 2013) with the FFT-NS-2 strategy, which was automatically selected using the –auto option. The aligned sequences were then used to construct a maximum-likelihood phylogenetic tree using IQ-TREE (v2.1.1) (Nguyen et al. 2015, Minh et al. 2020). The IQ-TREE used ModelFinder Plus (Kalyaanamoorthy et al. 2017) to automatically select the best-fit substitution model JJT+F+R5, and 1,000 ultrafast bootstrap replicates with UFBoot2 (Hoang et al. 2018) to assess branch support.

To determine whether the investigated *Methylobacter* genomes belong to the same genus, we calculated the average amino acid identity (AAI) using the EzAAI toolkit v1.2.2 (Kim et al. 2021), which extracts and estimates the pairwise similarity using Prodigal (Hyatt et al. 2010) and MMseqs2 (Steinegger and Söding 2017). *Methylobacter* species were delineated using the Type-Strain-Genome-Server (TYGS) (Meier-Kolthoff and Göker 2019, Meier-Kolthoff et al. 2022). This tool combines several indices and other types of evidence commonly used to distinguish species, including a phylogenomic tree calculated using genome BLAST distance phylogeny, digital DNA–DNA hybridization (dDDH), average nucleotide identity, and differences in genomic GC content. The dDDH scores were obtained using the Genome-to-Genome Distance Calculator 3.0, based on BLAST+ (Camacho et al. 2009) with the recommended formula 2 (d_4_) (Meier-Kolthoff et al. 2013, 2022), as well as the differences between genomic GC content (Meier-Kolthoff et al. 2014). Species were ultimately delineated with the agreement of all the used metrics, with dDDH being the conclusive one. Two genomes were clustered into the same species when the dDDH value >70% (Wayne et al. 1987, Goris et al. 2007), and into the same subspecies with >79% (Meier-Kolthoff et al. 2014, Meier-Kolthoff and Göker 2019).

We constructed phylogenetic trees with the 16S rRNA gene using nucleotide sequences and with protein-coding genes of interest using amino acid sequences (i.e. CH_4_ monooxygenases, ATP synthase subunit c, ATP synthases, and SoxB). In each case, sequences were aligned using MAFFT v7.526 with the –auto option to optimize the alignment process (Katoh and Standley 2013). The branch support in protein phylogenetic trees was obtained with the ultrafast bootstrap method with 1000 replicates (Hoang et al. 2018) implemented in the IQ-TREE 2 (Minh et al. 2020). Trees were visualized in iTOL v7 (Letunic and Bork 2024). Visualization of the genomic region of soluble CH_4_ monooxygenase was performed using the gggenomes package (Hackl et al. 2024) in R v4.0 (R Core Team 2024).

### Annotations of open reading frames

We screened all representative high-quality *Methylobacter* genomes and MAGs for the presence of genes encoding three CH_4_ monooxygenases and a gas vesicle protein with in-house HMMs with the hmmscan/hmmsearch function in HMMER 3.4 (Eddy 2011). The HMM for the major gas vesicle structural protein (encoded by the *gvpA* gene) was constructed from 32 reviewed and aligned amino acid sequences found in the UniProt Knowledgebase (The UniProt Consortium et al. 2025). All HMMER hits with a score <120 and an e-value <1e^−30^ were considered as *gvpA*. Additionally, we screened the genomes and MAGs for metabolic genes (i.e. [NiFe] hydrogenases, *soxB, narGHI*) using DIAMOND v2.1.9.163 (Buchfink et al. 2015) against the collections of reference amino acid sequences (Leung and Greening 2020). Sequences identified as [NiFe]-hydrogenases were subsequently classified into specific groups using HydDB (Søndergaard et al. 2016). Genes involved in iron metabolism were identified with HMMs from FeGenie (Garber et al. 2020). All MAGs were also annotated with the automatic pipeline DRAM (Shaffer et al. 2020). Additional confirmations of the annotations, i.e. for SoxB amino acid sequences, were obtained from InterPro scans (Blum et al. 2025).

To contextualize the distribution of genes encoding soluble methane monooxygenase (i.e. *mmoX)* and gas vesicles proteins (i.e. *gvpA)* among gammaproteobacterial methanotrophs, we downloaded 1,067 available genomes and MAGs listed as belonging to the order *Methylococcales* from NCBI on 12 August 2024, and used in-house HMMs to identify the presence of these genes.

Biosynthetic gene clusters (BCGs) were identified in the high-quality nonredundant genomes and MAGs with the Minimum Information about a Biosynthetic Gene cluster database v4.0 (Zdouc et al. 2025) using blastp in DIAMOND v2.1.9.163 (Buchfink et al. 2015), and antiSMASH v7.1.0 (Blin et al. 2023), both with stringent parameters.

### Predicting optimal growth conditions

Since the majority of *Methylobacter* species have not been cultured yet, little is known about their ecological preferences and optimal growth conditions. Therefore, we identified putative oxygen preferences, optimal temperature, salinity, and pH levels based on genomic amino acid compositions of all high-quality genomes and MAGs using GenomeSPOT with default statistical models (Barnum et al. 2024). The obtained predictions were compared with available data from cultured *Methylobacter* spp.

## Results and discussion

### Phylogenomic analysis and genome characteristics of *Methylobacter*

The *Methylobacter* genomes and MAGs differed substantially in general characteristics, such as size (3,452,370–5,467,791 bp), number of predicted proteins (3,206–5,043), and GC content (44%–55%) (Table S1, Fig. 1). The highest GC content occurred in the marine cluster and an adjacent cluster of *Methylobacter* spp. associated with eutrophic habitats and (putative) animal hosts (Table S1, Fig. 1). Accordingly, genomes that belong to psychrophilic strains and species from potentially nutrient-depleted habitats had the lowest GC content among all *Methylobacter* spp., reflecting adaptation to temperature and resource availability as described previously (Foerstner et al. 2005, Hu et al. 2022, Chuckran et al. 2023).

The genus *Methylobacter* contains two recognized, phylogenetic lineages, termed clade I, encompassing species such as *M. luteus* and *M. marinus*, and clade II, which includes the majority of *Methylobacter* spp. including *M. tundripaludum* (Smith et al. 2018). General AAI similarity recommended for clustering genomes/MAGs in one genus ranges between 65% and 95% (Konstantinidis et al. 2017); however, a *Methylobacter*-specific threshold has been set at 74% (Orata et al. 2018). The MAG *M. tundripaludum* Bin_16_1, which was the earliest diverging taxon, branched out most deeply in the phylogenomic tree (Fig. 1), shared only 72% AAI with all the genomes/MAGs in clade I, and 73%–74% AAI with the remaining genomes/MAGs, including its closest related *Methylobacter* genome, “*Ca*. Methylobacter favarea” (Fig. S1). Moreover, *M. tundripaludum* Bin_16_1 has been classified as belonging to the genus *Crenothrix* in the GTDB R10-RS226 (Parks et al. 2025) (Table S1). According to another measure of genome similarity allowing for genus delineation—a fixed percentage of conserved proteins set to 50%, clade II may actually consist of at least five separate genera (Orata et al. 2018), whereas the GTDB classification divides clade II into three genera: Methylobacter_A, Methylobacter_B, and Methylobacter_C in the GTDB R10-RS226 (Parks et al. 2025). However, ANIb values suggest that all the analysed genomes and MAGs, including *M. tundripaludum* Bin_16_1, belong to the genus *Methylobacter* based on the >73% ANI similarity threshold (Barco et al. 2020). Hence, our analyses suggest that the taxonomy of *Methylobacter*, and possibly that of *Crenothrix*, may need reevaluation, which is, however, beyond the scope of this study. Our phylogenomic analysis confirmed the separation of the two *Methylobacter* clades (Fig. 1). Additionally, we identified 33 unique species-level clusters based on several methods of species delineation, such as the TYGS clustering (Meier-Kolthoff and Göker 2019, Meier-Kolthoff et al. 2022), <95% of ANIb (Jain et al. 2018), and dDDH scores of <70 (Goris et al. 2007). Our analyses suggest that the genus *Methylobacter* may be composed of more species than previously recognized, with even more species to be identified in the near future through continued sequencing and culturing efforts.

We recently cultured two new *Methylobacter* spp., *Methylobacter* sp. Wu1 and *Methylobacter* sp. Wu8, from the sediment of a temperate eutrophic fishpond (Wutkowska and Daebeler 2024). The strain Wu1 shared >95% ANIb identity with “*Ca*. M. coli” BlB1 (Fig. S2), which was backed up by close clustering in the phylogenomic tree (Fig. 1). However, further evidence, i.e. a dDDH score of 64.3 in a pairwise genome comparison, suggested that they nevertheless likely belong to different species. Interestingly, “*Ca*. M. coli” BlB1 has been isolated from the faeces of a herbivore (Khatri et al. 2021), and it therefore seems plausible that *Methylobacter* sp. Wu1 may in fact originate from cattle manure, which is routinely used for fertilizing fishponds in the area (Potužák et al. 2007) and not from the sediment. Here, we tentatively propose the name *M. spei*, sp. nov. The genome of the Wu8 strain phylogenomically clustered most closely with three other MAGs of uncultured *Methylobacter* (Fig. 1). These were obtained from various aquatic environments in the northern hemisphere, and one of them, termed *M. tundripaludum* OWC-G53F, has previously been proposed to belong to a new species within the genus (Orata et al. 2018). Our ANIb and TYGS analyses corroborated the phylogenetic affiliation of the Wu8 strain with the uncharacterized species-level cluster distantly related to the next cultured relative (Fig. 1) and further suggested that it represents the first cultured member of a novel species for which we propose the name *M. methanoversatilis*, sp. nov. (Fig. 1; Fig. S2).

The phylogeny of the 18 available full-length 16S rRNA gene sequences from the analysed *Methylobacter* genomes was in congruence with the phylogenomic topology, preserving the major division into two clades (Fig. S3a). However, several distinct *Methylobacter* spp. displayed >99% nucleotide identity for the entire 16S rRNA gene (Khanongnuch et al. 2022, Rissanen et al. 2022), which is above the recommended 98.65% threshold to delineate bacterial species for this marker (Kim et al. 2014). For instance, the identity of the 16S rRNA genes of *M. psychrophilus* Z-0021 and *M. tundripaludum* SV96 is above this threshold, but their genome comparison yielded values below the 95% ANIb and 70% dDDH scores, which are recommended for species delineation (Goris et al. 2007, Jain et al. 2018), suggesting they are two distinct species. These discrepancies between the 16S rRNA gene-based and the genome-based species delineation can lead to erroneous conclusions when (often <250 bp) sequences of 16S rRNA generated in amplicon studies are used to study *Methylobacter* sp. community composition at the species level. Upon closer inspection, we also noticed that the most commonly targeted region of the 16S rRNA gene, the V4 region, which is also included in the Earth Microbiome Project (Caporaso et al. 2018), is highly conserved among several *Methylobacter* species. This was, for example, true for *M. tundripaludum* SV96 and *M. methanoversatilis* Wu8, which differed by only 1 nucleotide in the V4, but by 14 nucleotides in the V7–V8 region (Fig. S4). We therefore conclude that attempting to distinguish between *Methylobacter* species by comparing 16S rRNA gene sequences will often lead to an underestimation of diversity, as has been recently shown for other genera (Alleman et al. 2025). The PmoA-based trees (Fig. S3b and S3c) present a less congruent picture than the 16S rRNA and phylogenomic tree (Fig. S3a and Fig. 1, respectively), which is expected and has been previously shown to yield *Methylobacter* spp. as a polyphyletic genus (Knief 2015, Orata et al. 2018).

### A varied repertoire of CH_4_ and methanol oxidation

**Figure 2.**
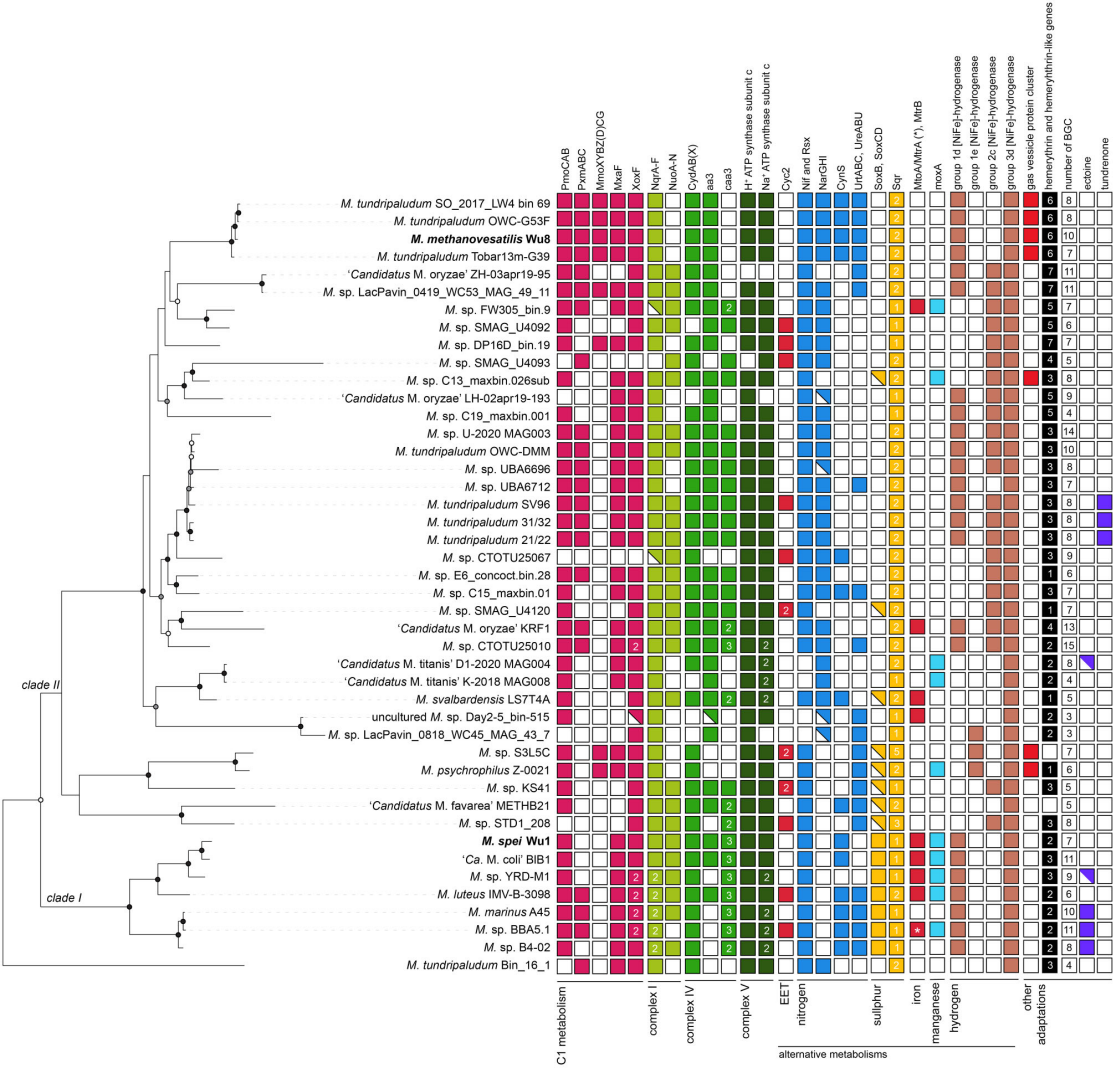
Distribution of distinct metabolic traits in *Methylobacter*. Protein homology was inferred based on the HMM models, alignment to collections of reference amino acid sequences, and automatic annotation using DRAM. Additionally, in some instances, we used phylogenetic trees to confirm functions and the identification of motifs determining specific functions (see the section “Materials and methods” for details). Solid, partial, and open squares indicate the presence, incompleteness, and absence of gene clusters, respectively. The name “*Methylobacter*” has been replaced by “*M*.”. Numbers inside the squares indicate the number of identified amino acid sequences. Abbreviations: EET: extracellular electron transport, PmoCAB: particulate methane monooxygenase (EC: 1.14.18.3); PxmABC: sequence-divergent particulate methane monooxygenase (EC: 1.14.18.3); MmoXYBZ(D)CG: soluble methane monooxygenase (EC: 1.14.13.25); MxaF: calcium-dependent methanol dehydrogenase (subunit 1; EC: 1.1.2.7); XoxF: lanthanide-dependent methanol dehydrogenase (EC: 1.1.2.10); NqrA-F: Na^+^-transporting NADH: ubiquinone oxidoreductase (EC: 7.2.1.1); NuoA-N: NADH: quinone dehydrogenase (EC: 7.1.1.2); CydAB(X): cytochrome bd(-I) (EC: 7.1.1.7); aa3: aa3-type cytochrome c oxidase with the adjacent hemerythrin (EC: 7.1.1.9); caa3: cytochrome c oxidase fused subunit I+III (characteristic for caa3-type cytochrome c oxidase) (EC: 7.1.1.9); Nif and Rsx: molybdenum-dependent nitrogenase complex (>10 genes found, EC: 1.18.6.1) and H^+^/Na^+^-translocating ferredoxin: NAD^+^ oxidoreductase (RsxABCDGE, EC: 7.1.1.11, 7.2.1.2); NarGHI: nitrate reductase (EC: 1.7.5.1, 1.7.99.-); CynS: cyanate lyase (EC: 4.2.1.104); UrtABC: urea transporter; UreABC: urease (EC: 3.5.1.5); SoxB: thiosulphohydrolase (EC: 3.12.1.1); SoxCD: *S*-disulphanyl-l-cysteine oxidoreductase (EC: 1.8.2.6); Sqr: sulphide: quinone oxidoreductase (EC: 1.8.5.4); Cyc2: putative iron oxidase that belongs to cluster 2 according to Garber et al. (2020); MtoA: decaheme c-type cytochrome according to Garber et al. (2020), MtrA(*) / MtrB: decaheme cytochrome, putatively involved in iron reduction; moxA: manganese oxidase (EC: 1.16.3.3) BGC: biosynthetic gene cluster. Genomes from our recently cultured strains are shown in bold.

**Figure 3.**
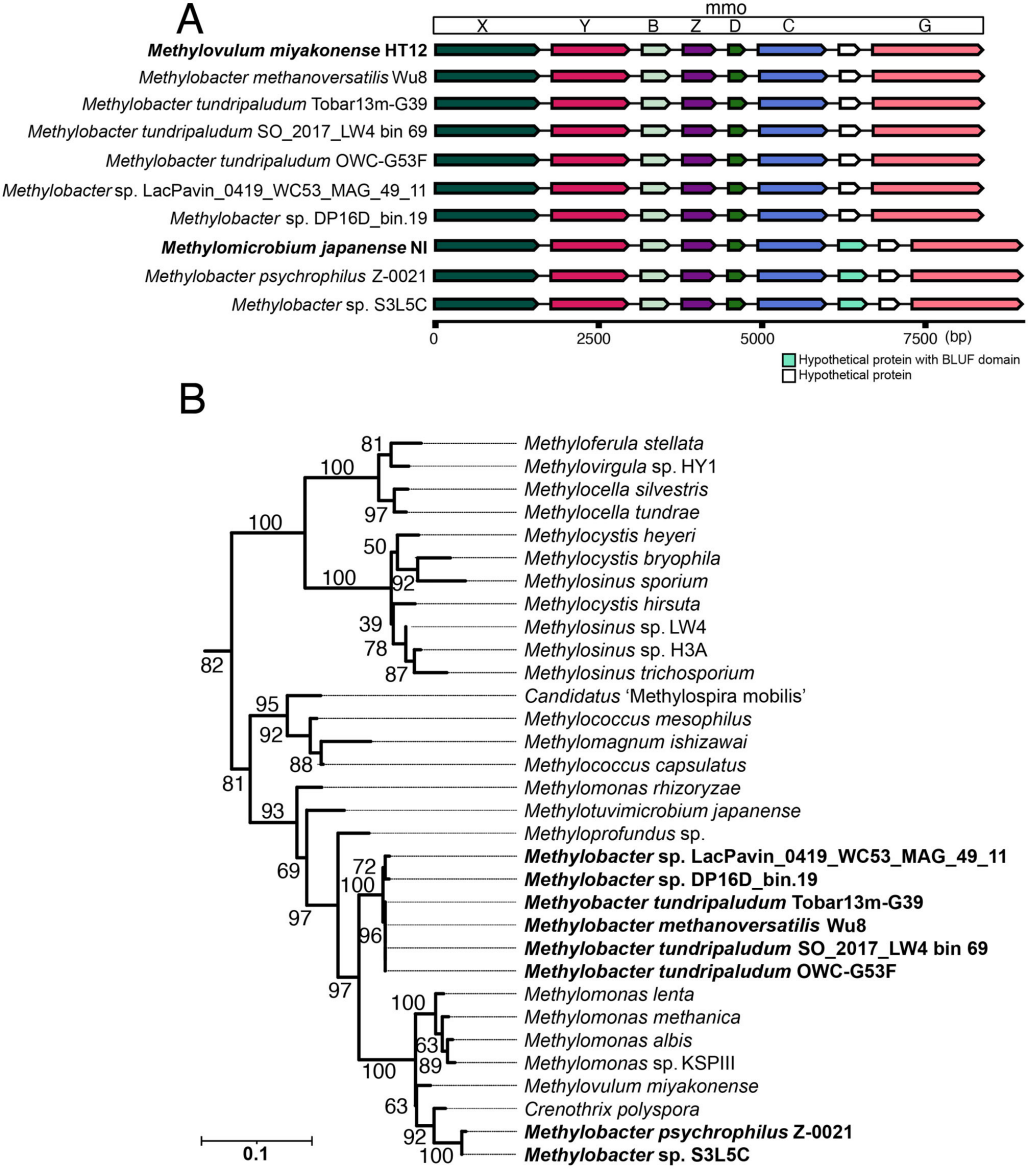
Genomic organization of the soluble CH_4_ monooxygenase operon and phylogeny of the MmoX subunit among methanotrophs. (A) Gene map of the sMMO operon across representative genomes, illustrating gene order and conservation. Gene lengths are calculated as the raw difference between end and start coordinates (in base pairs, bp), with small gaps added for visualization; the *x*-axis is therefore shown in bp. (B) Phylogenetic tree of the MmoX subunit from investigated *Methylobacter* spp. in the context of other methanotrophs from diverse taxonomic groups, showing taxonomic clustering patterns. Branch bootstrap support is indicated by numbers next to the nodes. The scale bar indicated 0.1 amino acid substitutions per site.

Nearly all the investigated *Methylobacter* genomes (91%) contained at least one gene cluster coding for particulate CH_4_ monooxygenase, either in its canonical *pmoCAB* (pMMO) or in the sequence-divergent *pxmABC* (pXMO) form (Tavormina et al. 2011) (Fig. 2). Almost half of the genomes (45%) encoded for both forms. Additionally, eight genomes (18%) contained the complete inventory for the cytoplasmic, soluble CH_4_ monooxygenase (sMMO) with structural (*mmoXYBZ(D)C*) and regulatory genes, such as *mmoG* (Figs 2 and 3). A phylogenetic analysis of the protein sequence of the hydrolase alpha subunit of sMMO, MmoX, showed a clear separation of *Methylobacter* MmoX into two clusters. MmoX sequences from *M. psychrophilus* Z-0021 and *Methylobacter* sp. S3L5C are affiliated closely with sequences from *Crenothrix polyspora* and *Methylovulum miyakonense* HT12, whereas the other cluster with the remaining MmoX, including sequences from *M. methanoversatilis*, formed a deeper branching, isolated group (Fig. 3B). The latter cluster lost the additional gene coding for an unknown hypothetical protein between *mmoC* and *mmoG*, similarly to *M. miyakonense* HT12 (Iguchi et al. 2010) (Fig. 3A and B).

Although the presence of sMMO has been reported for the genomes of psychrophilic *M. psychrophilus* Z-0021 (Rissanen et al. 2022) and in *Methylobacter* sp. S3L5C (Khanongnuch et al. 2022), earlier works had failed to identify sMMO in *M. psychrophilus* (Omelchenko et al. 1996, Trotsenko and Khmelenina 2005). The presence of sMMO is rarely associated with *Methylobacter* spp. (Bowman et al. 1993, Smith et al. 1997, Bowman 2014), or in fact, with the majority of the *Methylococcales* (Semrau 2011, Dedysh and Knief 2018). However, in our survey of >1,000 genomes of *Methylococcales*, MmoX was found in ∼15%, belonging to *Methylococcus, Crenothrix, Methylomonas, Methyloprofundus, Methylovulum, Methylomagnum*, “*Ca*. Methylocalor cossyra,” “*Ca*. Methylospira mobilis,” and many uncultured *Methylococcales* (Table S2).

Interestingly, all four strains belonging to the cluster with *M. methanoversatilis* encoded all three CH_4_ monooxygenase gene clusters: pMMO, pXMO, and sMMO (Fig. 2). Although uncommon, the occurrence of all three MMOs has been reported for other methanotrophs, including *Methylocystis hirsuta* CSC1, *Methylocystis bryophila* S285, and *Methylosinus* sp. R-45379 (Han et al. 2018, Oshkin et al. 2020). Despite its rare occurrence, the presence of all three MMO forms might be an ancestral trait, as all three MMOs have been inferred to be present in extant ancestral proteobacterial methanotrophs (Osborne and Haritos 2018). Likely, this repertoire confers broader metabolic flexibility for energy conservation from CH_4_ oxidation by providing different substrate affinities and specificities (Sullivan et al. 1998, Baani and Liesack 2008).

The metabolic flexibility of *Methylobacter* spp. is also evident in their genomic repertoire for the second step of CH_4_ oxidation—the oxidation of methanol to formaldehyde. The majority of analysed genomes encoded for calcium-dependent and lanthanide-dependent methanol dehydrogenase, *mxaFI-MDH* (EC: 1.1.2.7) and *xoxF-MDH* (EC: 1.1.2.10), respectively. The latter contains only one subunit, which was found in almost all analysed *Methylobacter* genomes as a single-copy gene (84% of genomes) or with two copies (11% of genomes) (Fig. 2, Table S3). Xox-MDH can act as a primary methanol dehydrogenase (Chu and Lidstrom 2016) and has been speculated to be more efficient and potentially more metabolically versatile than the calcium-dependent form, for instance, by not only being able to oxidize methanol to formaldehyde but also to formate (Keltjens et al. 2014). Formaldehyde can be assimilated into biomass through the ribulose-5-phosphate (RuMP) cycle or converted to formate via the tetrahydromethanopterin (H_4_MPT) pathway, which can subsequently be converted into CO_2_ with formate dehydrogenase.

### Versatile electron transport chain

Our comparative genomics analyses revealed several variations of electron transport chain components in *Methylobacter*, which are likely adaptations to diverse habitats and provide the capacity to withstand fluctuating environmental conditions. First, we identified redox-driven Na^+^-transporting NADH: ubiquinone oxidoreductase (EC: 7.2.1.1; NQR encoded by *nqrA–F*), which acts as a complex I, in almost all analysed *Methylobacter* spp. (Fig. 2, Table S3). Several genomes in clade I, including those of marine species, encoded two NQRs. Another type of complex I, NADH: quinone dehydrogenase (EC: 7.1.1.2, NUO encoded by *nuoA–N*) was present in the majority of the genomes, but lacking in many *Methylobacter* genomes and MAGs, including the four strains of *M. methanoversatilis* (Fig. 2, Table S3). Both forms of complex I transfer an electron from NADH to quinone, but they pump different cargo to the periplasm: either two protons (NUO) or one Na^+^ ion (NQR); however, it has been shown that NQR can also translocate protons (Raba et al. 2018). Moreover, these complexes differ in their energy conservation capacity, with NQR being likely more energy-efficient compared to NUO (Hreha et al. 2021). A fine-tuned differential expression of these two forms has been observed in *M. tundripaludum* SV96, with the NQR dominating expression at temperatures below 15°C (Tveit et al. 2023). Additionally, it is likely that the expression of NQR/NUO may be regulated by other factors, such as stoichiometry of Na^+^/e^−^ and H^+^/e^−^, redox, and low O_2_ levels and substrate availability, as indicated for other bacteria (Bogachev et al. 1997, Spero et al. 2015, Ito et al. 2020, Kaila and Wikström 2021). Certainly, the possibility to choose between them allows those *Methylobacter* spp. that possess both forms to make optimal use of resources for maintaining and building membrane potential. Sole reliance on NQR, on the other hand, may reflect adaptation to elevated salt concentration and/or be necessary for anaerobic metabolism (Buckel et al. 2025).

Secondly, we detected up to two high-affinity terminal oxidases of the *bd(-I)*-type (EC: 7.1.1.7; Fig. 2) in the vast majority of investigated *Methylobacter* genomes. These cytochrome *bd* quinol oxidases consist either of two or three subunits (genes *cydAB(X)*). They have been found to enable aerobic respiration under hypoxia (<50 nM O_2_) and withstand nitrosative and oxidative stress better than other types of terminal oxidases (Giuffrè et al. 2014). Next to these *bd*-type oxidases, most analysed *Methylobacter* spp. contained other terminal oxidases of the heme-copper A and C types in their genomes (Fig. 2, Table S3). In all genomes containing the *aa3*-type terminal oxidase, which has a low affinity for O_2_ (Berg et al. 2022), we found a gene coding for hemerythrin adjacently located, as seen in other gammaproteobacterial methanotrophs (Rahalkar and Bahulikar 2018, Nariya and Kalyuzhnaya 2020, Weiblen et al. 2025). Hemerythrins act as high-affinity O_2_-sensors and carriers, whose expression in methanotrophs has been linked to hypoxia (Kalyuzhnaya et al. 2013, Rahalkar and Bahulikar 2018, Nariya and Kalyuzhnaya 2020, Weiblen et al. 2025) and to high copper concentrations with a pMMO-enhancing function (Kao et al. 2008, Chen et al. 2012). Depending on the oxygen availability, the transcription of hemerythrin and terminal oxidase genes may differ; however, they seem to work together to aid optimal respiration for the given conditions (Nariya and Kalyuzhnaya 2020). Since hemerythrins enhancedO_2_ flux under O_2_-limited conditions in *Methylomicrobium alcaliphilum* (Nariya and Kalyuzhnaya 2020), it is possible that the exact mechanism can occur in *Methylobacter* spp.

Finally, we identified the presence of Na⁺-translocating ATP synthases next to the H^+^-translocating ones in most analysed genomes (Fig. 2, Table S3). By comparing the amino acid sequences of the subunit c, encoded by *atpE*, from the Na⁺-translocating ATP synthases, we found subunits with one- and two-carboxylate ion coupling motifs (Fig. S5B). The two-carboxylate form has been shown to enable the enzyme to couple Na^+^ transport to ATP synthesis only when Na^+^ is in excess over H^+^ in the environment (Schulz et al. 2013, Leone et al. 2015). This diversity in components of the respiratory chain, together with the presence of diverse dissimilatory protein complexes (see below), most likely allows *Methylobacter* spp. to employ flexible mechanisms suited for thriving in different, often fluctuating, environmental conditions.

### Alternative metabolisms

Our analyses revealed a broad genomic potential of *Methylobacter* spp. to use various alternative electron acceptors. For instance, we identified genes involved in partial denitrification in the majority of the analysed clade II genomes and MAGs. Many of the investigated *Methylobacter* spp. have the potential to reduce nitrate to nitrite [e.g. with the dissimilatory nitrate reductase (EC: 1.7.5.1, 1.7.99.-)], nitrite to nitric oxide [e.g. with the nitrite reductase (NO-forming) (EC: 1.7.2.1)], and nitric oxide to nitrous oxide [e.g. cytochrome c coupled nitric oxide reductase (EC:1.7.2.5)] (Fig. 2, Table S3). However, we did not detect nitrous oxide reductase encoded by *nosZ* reported for some acidophilic methanotrophs belonging to *Alphaproteobacteria* and *Verrucomicrobiia* (Awala et al. 2024). Indeed, axenic cultures of *Methylobacter* sp. YRD-M1 growing in O_2_-limiting conditions produced N_2_O or NO (Hao et al. 2022). Previous studies showed that dissimilatory nitrate reduction enabled aerobic CH_4_ oxidation under O_2_-limited conditions, with nitrate as an electron acceptor, in *Methylomonas denitrificans* (Kits et al. 2015). *Methylobacter* spp. from cluster II, possess the essential genes for this pathway (*narGHI*; Fig. 2) and have been shown to carry out this process in similar conditions (Li et al. 2025).

We have also identified genes encoding potential alternative metabolisms that use metal (oxyhydr)oxides as electron donors and acceptors, for instance, iron oxides, ferrihydrite, and manganese (Fig. 2). Especially genes involved in iron metabolism and extracellular electron transport, have been linked to CH_4_ oxidation in O_2_-limited and -depleted conditions (Zheng et al. 2020, Li et al. 2023, 2024), also in sediments with *Methylobacter* spp. (Vigderovich et al. 2023).

Our analyses furthermore revealed that sulphide: quinone oxidoreductase (*sqr*; EC: 1.8.5.4) was universally present in all 44 *Methylobacter* genomes and MAGs in one or more copies (Fig. 2, Table S3). Moreover, we detected SoxB [thiosulphohydrolase/*S*-sulphosulphanyl-l-cysteine sulphohydrolase (EC: 3.12.1.1)] in several *Methylobacter* spp. genomes and MAGs (Fig. 2; Fig. S6, Table S3). The SoxB sequences identified in our study were phylogenetically grouped into two clusters: a cluster with seven sequences identified by search against curated databases (see the section “Methods” for details) and an additional cluster with eight sequences identified by automatic annotations (the automatic pipeline found all 15 sequences). The presence of sequences from the first cluster coincided with the presence of additional genes from the thiosulphate-oxidizing complex encoding SoxC and SoxD [*S*-disulphanyl-l-cysteine oxidoreductase (SoxCD; EC: 1.8.2.6)] comprising components of the periplasmic thiosulphate-oxidizing complex catalysing thiosulphate oxidation to sulphate (Fig. 2, Table S3). However, in the genomes with the sequences from the second cluster *soxC* and *soxD* were missing (Fig. 2). However, a recent study showed that *Methylobacter* sp. S3L5C, which encodes only a SoxB and not SoxCD, can nevertheless use reduced sulphur compounds, such as sulphide, and dissolved organic matter as electron donors, which enabled elevated methane oxidation rates and higher biomass production (Rissanen et al. 2025a). During this process, *Methylobacter* sp. S3L5C maintained elevated expression of *sqr* and *soxB*.

According to our analyses, all *Methylobacter* genomes contained [NiFe]-hydrogenases of the 1d, 1e, 2c, and 3d groups in various combinations (Fig. 2, Table S3). The groups 1d and 1e are known to be involved in respiratory hydrogen uptake liberating electrons for aerobic and anaerobic respiration (1d) or sulfur respiration (1e), whereas hydrogenases of the group 2c and 3d on the other hand are cytosolic and not known to be coupled to energy conservation (Vignais et al. 2001, Greening et al. 2016, Søndergaard et al. 2016). Only the hydrogenase of the 3d group was present in all analysed *Methylobacter* genomes and MAGs. They most likely act as redox valves, either providing NADH as a reductant for carbon fixation or catalysing the fermentative production of hydrogen (Vignais et al. 2001, Søndergaard et al. 2016). Despite this apparent diversity of hydrogenases present in *Methylobacter* genomes and MAGs, to our knowledge, there is no evidence on the metabolic use of hydrogen from axenically cultured *Methylobacter* spp. This contrasts with the proven hydrogen utilization by alphaproteobacterial (Hakobyan and Liesack 2020, Hakobyan et al. 2020), verrucomicrobial (Carere et al. 2019), and gammaproteobacterial methanotrophs with the Calvin–Benson–Bassham cycle (Stanley and Dalton 1982, Liu et al. 2024). However, anaerobic laboratory incubations of complex microbial communities from Arctic lake sediments revealed the expression of *Methylobacter* group 1d hydrogenases, suggesting that hydrogen may provide energy for CH_4_ oxidation by *Methylobacter* spp. under O_2_-depleted conditions (He et al. 2022).

**Figure 4.**
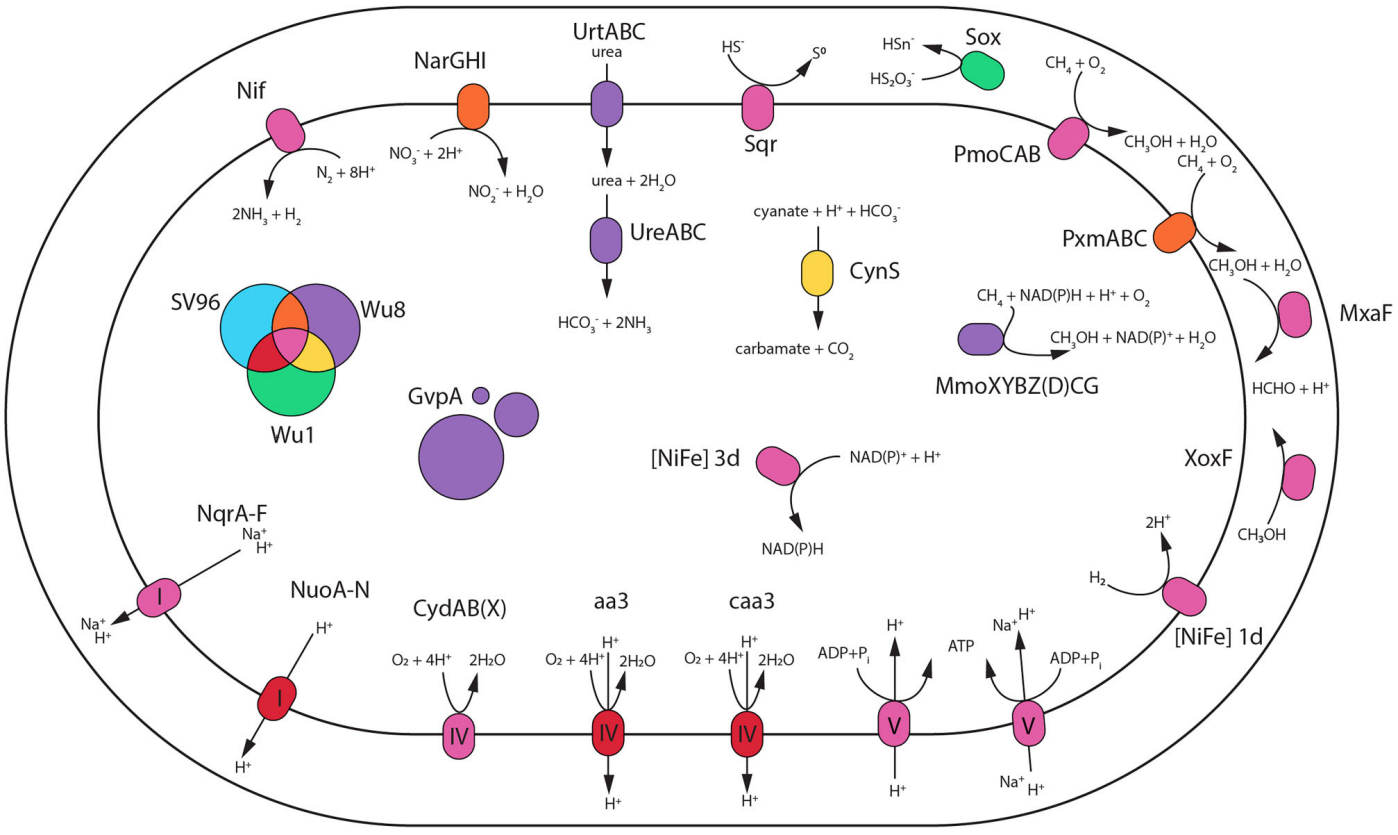
Visual depiction of some of the proteins and protein complexes in three *Methylobacter* spp.: *M. tundripaludum* SV96, *M. methanoversatilis* Wu8, and *M. spei* Wu1. Abbreviations: PmoCAB: particulate methane monooxygenase (EC: 1.14.18.3); PxmABC: sequence-divergent particulate methane monooxygenase (EC: 1.14.18.3); MmoXYBZ(D)CG: soluble methane monooxygenase (EC: 1.14.13.25); MxaF: calcium-dependent methanol dehydrogenase (subunit 1; EC: 1.1.2.7); XoxF: lanthanide-dependent methanol dehydrogenase (EC: 1.1.2.10); NqrA–F: Na^+^-transporting NADH: ubiquinone oxidoreductase (EC: 7.2.1.1); NuoA–N: NADH: quinone dehydrogenase (EC: 7.1.1.2); CydAB(X): cytochrome bd(-I) (EC: 7.1.1.7); aa3: aa3-type cytochrome c oxidase (EC: 7.1.1.9); caa3: cytochrome c oxidase fused subunit I+III (characteristic for caa3-type cytochrome c oxidase) (EC: 7.1.1.9); Nif: molybdenum-dependent nitrogenase complex (>10 genes found, EC: 1.18.6.1); NarGHI: nitrate reductase (EC: 1.7.5.1, 1.7.99.-); CynS: cyanate lyase (EC: 4.2.1.104); UrtABC: urea transporter; UreABC: urease (EC: 3.5.1.5); Sox: potential for oxidizing sulphur compounds, such as thiosulphate.

These different genes may play important roles in utilizing alternative electron donors and acceptors; however, especially the hydrogen- and sulfur-related genes and pathways are understudied in *Methylobacter* spp. (Fig. 4), and largely in methanotrophs in general. Therefore, it is unclear how much they contribute to energy generation relative to CH_4_ oxidation, and under which conditions these metabolisms could be relevant to the cells. Moreover, our analyses do not provide a complete list of putative electron donors and acceptors.

### Diverse genomic potential for nitrogen fixation and assimilation

*Methylobacter* spp. are known for their diverse roles in the nitrogen cycle (Bowman et al. 1993, Bowman 2014, Khatri et al. 2020). We identified at least 10 genes for molybdenum-dependent nitrogenase (Nif; Fig. 2, Table S3). The presence of Nif coincided with the presence of a gene cluster coding for H^+^/Na^+^-translocating ferredoxin: NAD^+^ oxidoreductase (*rsxABCDGE*, EC: 7.1.1.11, 7.2.1.2), which is architecturally similar to NQR, or Rnf gene cluster found in anaerobic organisms (Koo et al. 2003). Rsx transfers electrons to a transcriptional regulator involved in responding to oxidative and nitrosative stress—SoxR, which is reduced during aerobic growth (Ding and Demple 1997, Koo et al. 2003). Aerobic dinitrogen fixation has been demonstrated for the Nif and Rsx-containing “*Ca*. M. svalbardensis” (Patil et al. 2024), and it is therefore probable that the other *Methylobacter* spp. with the same gene clusters are capable of this metabolism.

*Methylobacter* spp. isolated from various environments can assimilate both nitrate (e.g. with *nasAB*) and ammonia (e.g. with *glnA, gdhA*) as they can grow in both nitrate- and ammonia-amended salt media (Bowman et al. 1993, Bowman 2014). Additionally, we found that more than half of the analysed species held the potential for assimilation of (reduced) forms of dissolved organic nitrogen, such as cyanate and/or urea (Fig. 2, Table S3), possibly also ammonia, as we identified genes encoding for hydroxylamine detoxification (Table S3). Urea has been identified as a source of nitrogen for several methanotrophs (de la Torre et al. 2015, Nguyen et al. 2017, Wang et al. 2024), and it stimulated the growth of *Methylobacter*-like species (Zheng et al. 2014). “*Ca*. Methylobacter coli” strain BlB1 has been shown to grow on urea as a source of nitrogen (Khatri et al. 2021); however, in our analyses, we have identified neither urease nor urea-transporting genes (Fig. 2). Moreover, urease may play additional roles, for instance, in the adjustment of internal or external pH (Scott et al. 1998, Stingl et al. 2002). For other organisms, it has been demonstrated that cyanate serves as a nitrogen source, facilitating growth (Guilloton and Karst 1987, Wood et al. 1998). Clearly, however, organic nitrogen uptake by *Methylobacter* spp., and perhaps by all methanotrophs, remains functionally unexplored and understudied.

### Unusual genomic adaptations

Unexpectedly, we found a multigene cluster encoding gas vesicle proteins in all four members of *M. methanoversatilis*, as well as the two psychrophilic species *M. psychrophilus* Z-0021 and *Methylobacter* sp. S3L5C, and a groundwater MAG that belonged to *Methylobacter* sp. C13 (Fig. 2, Table S3). Typically, the gas vesicles protein gene clusters contain 8–14 genes, and produce a vesicle protein monolayer formed by the gas vesicle protein A (encoded by *gvpA*). The presence of this gene cluster suggests a planktonic lifestyle, as gas vesicles are typically known for enabling organisms to control their buoyancy in the water column (Walsby 1994, Pfeifer 2012). The protein gas vesicles have only been reported for three methanotrophs so far—“*Ca*. Methylomirabilis limnetica” known for its planktonic lifestyle (Graf et al. 2018), *Methylosphaera hansonii* isolated from the hypolimnion of an Antarctic lake (Bowman 2015), and *M. psychrophilus* Z-0021 isolated from tundra soil (Omelchenko et al. 1993). To place our findings in a wider context for gammaproteobacterial methanotrophs, we surveyed >1,000 genomes and MAGs of *Methylococcales* and detected at least one *gvpA* gene copy in ∼12.5% of them (Table S2). Interestingly, they were present in up to five copies of *gvpA*, primarily in the MAGs of uncultured *Methylococcaceae* (NCBI-sourced taxonomy) from O_2_-stratified freshwater bodies in the Northern Hemisphere sequenced in Buck et al. (2021). As some methanotrophs are known to grow preferentially in defined CH_4_-O_2_ gradients (Bussmann et al. 2006, Reim et al. 2012, Danilova et al. 2016, Beals and Puri 2024), perhaps the ability to regulate buoyancy enables them to position themselves at the water column depths with optimal CH_4_:O_2_ stoichiometry. However, some of the *Methylobacter* strains in which we detected *gvpA* originate from terrestrial habitats (Fig. 1, Table S1). Other terrestrial bacteria have been reported to contain *gvp* gene clusters (Van Keulen et al. 2005), and the function of these organelles remains little understood. Gas vesicles increase the cell surface-to-volume ratio and therefore they can improve gas diffusion and aid in survival under stressful conditions (Walsby 1972, 1994, Pfeifer 2012). Indeed, *M. psychrophilus Z-0021* has been shown to produce gas vesicles increasingly with temperatures ranging from 7°C to 20°C, but no gas vesicles were detected when the temperature dropped below 7°C (Omelchenko et al. 1993). These findings imply that increased gas vesicle production helps to overcome substrate limitation due to lower CH_4_ and O_2_ solubility in the medium with increasing temperature. Protein vesicles are permeable to many gases, including CH_4_ and O_2_ (Walsby 1969, 1971, 1982), but it is unclear which gas(es) methanotrophs contain in the vesicles. It is tempting to speculate that the accumulated gas(es) could serve as a temporal reservoir of metabolically important gases, such as O_2_ or CH_4_. However, these speculations require appropriate experimental verification.

On average, we identified nearly eight BGCs per analysed *Methylobacter* genome/MAG, ranging from three in the genomes of organisms from nutrient-poor, pelagic environments to 15 in a MAG from freshwater sediment (Fig. 2). The most attention among *Methylobacter* spp. BGCs was given to tundrenone, a quorum-sensing molecule likely involved in hypoxia stress response (Puri et al. 2018, Yu et al. 2020), and to ectoine, an organic osmoprotectant that, among other functions, increases halotolerance (Reshetnikov et al. 2011). Nevertheless, we could only confirm the complete tundrenone gene cluster (encoded by *tunA–P* and several regulatory genes on both sides of the gene cluster) for *M. tundripaludum* SV96, 21/22, and 31/32 (Fig. 2). Similarly, a complete ectoine BGC was only detected in three genomes of the clade I: *M. marinus* A45, *Methylobacter* sp. BBA5.1, and *Methylobacter* sp. B4-02 (Fig. 2). Moreover, most genomes/MAGs contained more than one BGC encoding terpenes, a metabolite group involved in communication or interaction between species, that has been shown to be produced by *M. luteus* in the presence of *Pseudomonas mandellii* (Veraart et al. 2018). Furthermore, all analysed *Methylobacter* contained at least one BGC for redox-cofactors and aryl polyenes. The latter have recently been reported as common in gammaproteobacterial methanotrophs (Krause et al. 2025) and may encode antioxidative pigments (Schöner et al. 2016). Finally, we detected an abundance of diverse, yet unexplored, BGC gene clusters encoding for polyketides, which are known to often carry antibiotic and pharmacological properties. Impressively, two BGCs contained polyketide synthetases that were among the longest genes in *M. methanoversatilis*, spanning over 18.5 kbp (NCBI Reference Sequence: WP_331306173.1) and 26 kbp (NCBI Reference Sequence: WP_331307029.1). Despite the availability of genomic information and the presence of gene clusters with high interest for applied science, the biotechnological potential of *Methylobacter* spp. has not been fully explored. Genomic data can be used for metabolic modeling (Islam et al. 2020, Wutkowska et al. 2024) to describe the functioning of *Methylobacter* spp. alone and within microbial communities, which may direct future design of biotechnological applications.

### Genome-inferred optimal growth conditions may guide future cultivation efforts

New strains of methanotrophs are being isolated and described, i.e. new *Methylobacter* spp. (Risannen et al., 2025b). However, a large diversity of methanotrophs, including most analysed *Methylobacter* spp., remains uncultured, which hampers the investigation of genome-derived hypotheses regarding their metabolism and ecological niche. To assist and possibly direct new cultivation efforts, we predicted optimal conditions for growth regarding temperature, salinity, and pH, as well as tolerance to oxygen from the 44 high-quality genomes and MAGs (Fig. S7). Comparing the predictions with experimentally verified optima for those species that are cultured showed that the genome-wide amino acid composition predictions do not identify the exact optima (Fig. S7). The general temperature and salinity preferences were likely accurately predicted; for example, the predicted temperature optima for known psychrophiles were among the lowest (Fig. S7a), and known halophilic species and species from oligotrophic environments were predicted to fall at opposite ends of the salinity range (Fig. S7c). Contrastingly, the predictions of optimal pH failed to confirm known pH preferences (Fig. S7b) and were associated with large uncertainties. Without further studies, it is challenging to identify the cause of these discrepancies.

Although all *Methylobacter* genomes and MAGs in this study were expectedly tagged as oxygen-tolerant and are classified as aerobic CH_4_ oxidizers, an increasing number of studies reported *Methylobacter* spp. found in O_2_-limited or O_2_-depleted environments (van Grinsven et al. 2020b, Nercessian et al. 2005, Biderre-Petit et al. 2011, Graef et al. 2011, Reim et al. 2012, Hernandez et al. 2015, Oshkin et al. 2015, Martinez-Cruz et al. 2017, Rissanen et al. 2018, Singleton et al. 2018, Cabrol et al. 2020, Mayr et al. 2020, 2020a, Hao et al. 2022, He et al. 2022, Grégoire et al. 2023, Li et al. 2023, 2024, Gafni et al. 2024, Kallistova et al. 2024, Reis et al. 2024, Schorn et al. 2024, 2025), and sometimes able to outcompete other methanotrophs (Beck et al. 2013, Hernandez et al. 2015, Islam et al. 2020, Li et al. 2025). To date their persistence or continuous activity under O_2_-limited and O_2_-depleted conditions is far from being fully understood (Hernandez et al. 2015, Gafni et al. 2024, Li et al. 2024, Reis et al. 2024, Ruff et al. 2024), but in the analysed *Methylobacter* genomes and MAGs we found many genes that provide insights into their functioning under O_2_-depleted conditions, such as nitrate reductases, high-affinity terminal oxidases, hemerythrins, hydrogen uptake [NiFe]-hydrogenases, and the inventory for sulphide and thiosulphate oxidation (Fig. 2, Table S3). Evidence for additional genes responding to O_2_-depleted conditions is being continuously reported. For instance, a recent transcriptomics study found that some proteins within the type VI secretion system operon, which are present only in genomes of the clade I, including *M. luteus*, were upregulated in low O_2_ conditions (Beals and Puri 2024). Attempting to culture and isolate new *Methylobacter* spp. in carefully designed O_2_-depleted conditions, therefore, represents a promising avenue to a better understanding of their spread in the environment.

## Conclusions

Despite apparent environmental importance and widespread occurrence, *Methylobacter* species are still poorly understood in many aspects. Our study shows that the genus harbors more species than currently described, and some of them cannot be distinguished based on 16S rRNA gene sequence comparison. *Methylobacter* species display diverse adaptations to their environments, i.e. diverse combinations of the three different CH_4_ monooxygenases and the potential for several dissimilatory metabolisms. We would also like to encourage testing some of the metagenomic-based hypotheses that we outlined here to move to the postgenomic era with the *Methylobacter* spp.

## Taxonomic considerations

### Description of *Methylobacter spei*, sp. nov.

Etymology: L. fem. n. *spes*, hope; genitive singular *spei*, meaning “of hope,” since the organism was enriched from a fishpond in SE Czech Republic called Hope (Naděje).

A freshwater aerobic chemoorganotroph that oxidizes CH_4_ was obtained from organic-rich sediment in an eutrophic fishpond near Hluboká nad Vltavou, called Hope (Naděje). Phylogenetically affiliated with the genus *Methylobacter*, family *Methylomonadaceae*, order *Methylococcales*, phylum *Pseudomonadota*. The genome is 100% complete with 0.36% contamination (CheckM2). It consists of 85 contigs of a total length of 4.4 Mbp and 52% GC content. The coding density is 86.5% with 4,078 total predicted protein sequences and an average gene length of 311.5 bp.

The genome Wu1^Ts^ represented by a MAG (GenBank accession numbers: GCA_036553575.1 and GCF_036553575.1), is designated the nomenclatural type for the species, and was recovered from a low-complexity enrichment containing three species.

### Description of *Methylobacter methanoversatilis*, sp. nov.

Etymology: *methyloversatilis* N.L. neut. n. *methanum*, methane; L. adj. *versatilis*, versatile, adaptable; N.L. adj. *methanoversatilis*, referring to the potential metabolic versatility of the organism in utilizing CH_4_ with three diverse CH_4_ monooxygenases, i.e. pMMO, pXMO, and sMMO, encoded in the genomes.

A freshwater aerobic chemoorganotroph that oxidizes CH_4_ was obtained from organic-rich sediment of a eutrophic fishpond in the vicinity of Hluboká nad Vltavou called Hope (Naděje). Phylogenetically affiliated with the genus *Methylobacter*, family *Methylomonadaceae*, order *Methylococcales*, phylum *Pseudomonadota*. The proposed species is represented by 4 MAGs: *Methylobacter tundripaludum* OWC-G53F (GenBank accession numbers: GCA_002934365.1 and GCF_002934365.1), *Methylobacter tundripaludum* Tobar13m-G39 (GenBank accession number: GCA_021736725.1), *Methylobacter tundripaludum* SO_2017_LW4 bin 69 (GenBank accession number: GCA_023227865.1), *Methylobacter* sp. Wu8 (GenBank accession numbers: GCA_036440755.1 and GCF_036440755.1), collected in four different geographic localities. The latter was obtained from an axenic culture that is no longer available. None of the genomes are complete/circular, however, they are of high quality and low contamination (Table 1). The genome size is 4.03–4.18 Mbp, with the G+C content 51%–52%, coding density 88.1%–88.3%, total predicted coding sequences 3,715–3,788, and average gene length 319.5–327.7 bp. The metabolic predictions indicated that the organism encodes for three different CH_4_ monooxygenases, i.e. two particulate (pMMO and pXMO) and soluble (sMMO).

**Table 1.** Characteristics of four *Methylobacter* spp. genomes that formed a cluster together with the *M. methanoversatilis* strain Wu8.

GenBank assembly	Size (Mbp)	Completeness (%)	Contamination (%)	Coding density (%)	Number of encoded proteins	Average gene length (bp)	Sample origin
GCA_002934365.1	4.18	99.92	0.00	88.10	3,754	327.7	USA
GCA_021736725.1	4.05	99.86	0.01	88.30	3,719	320.6	Spain
GCA_023227865.1	4.03	91.74	2.29	88.20	3,715	319.5	USA
GCA_036440755.1	4.16	95.92	0.00	88.10	3,788	322.8	Czechia

The genome Wu8^Ts^ represented by a MAG available under the GenBank accession number: GCA_036440755.1, is designated nomenclatural type for the species, and was recovered from temperate eutrophic fishpond sediments.

## Supplementary Material

fiaf127_Supplemental_Files

## Data Availability

The data were derived from sources in the public domain. 97 *Methylobacter* spp. genomes and MAGs were downloaded from NCBI Genome portal on May 21, 2024, from https://www.ncbi.nlm.nih.gov/datasets/genome/?taxon=429. 1067 *Methylococcaceae* genomes and MAGs were downloaded from NCBI Genome portal on August 12, 2024, from https://www.ncbi.nlm.nih.gov/datasets/genome/?taxon=403. Code generated to analyse the data is deposited at https://github.com/magdawutkowska/methylobacter_comparative_genomics/. The names of the novel *Methylobacter* species *Methylobacter methanoversatilis*, sp. nov. and *Methylobacter spei*, sp. nov. have been registered under the SeqCode: https://seqco.de/i:52928 and https://seqco.de/i:52927, respectively.

## References

[bib1] Alleman AB, Stolyar S, Marx CJ et al. Led astray by 16S rRNA: phylogenomics reaffirms the monophyly of *Methylobacterium* and lack of support for *Methylorubrum* as a genus. ISME J. 2025;19:wraf011. 10.1093/ismejo/wraf011. 39834026 PMC11833323

[bib2] Awala SI, Gwak J-H, Kim Y et al. Nitrous oxide respiration in acidophilic methanotrophs. Nat Commun. 2024;15:4226. 10.1038/s41467-024-48161-z. 38762502 PMC11102522

[bib3] Baani M, Liesack W. Two isozymes of particulate methane monooxygenase with different methane oxidation kinetics are found in *Methylocystis* sp. strain SC2. Proc Natl Acad Sci USA. 2008;105:10203–8. 10.1073/pnas.0702643105. 18632585 PMC2481331

[bib4] Barco RA, Garrity GM, Scott JJ et al. A genus definition for *Bacteria* and *Archaea* based on a standard genome relatedness index. mBio. 2020;11:e02475–19. 10.1128/mBio.02475-19. 31937639 PMC6960282

[bib5] Barnum TP, Crits-Christoph A, Molla M et al. Predicting microbial growth conditions from amino acid composition. bioRxiv. 2024. 10.1101/2024.03.22.586313

[bib7] Beals DG, Puri AW. Linking methanotroph phenotypes to genotypes using a simple spatially resolved model ecosystem. ISME J. 2024;18:wrae060. 10.1093/ismejo/wrae060. 38622932 PMC11072679

[bib8] Beck DAC, Kalyuzhnaya MG, Malfatti S et al. A metagenomic insight into freshwater methane-utilizing communities and evidence for cooperation between the *Methylococcaceae* and the *Methylophilaceae*. PeerJ. 2013;1:e23. 10.7717/peerj.23. 23638358 PMC3628875

[bib9] Berg JS, Ahmerkamp S, Pjevac P et al. How low can they go? Aerobic respiration by microorganisms under apparent anoxia. FEMS Microbiol Rev. 2022;46:fuac006. 10.1093/femsre/fuac006. 35094062 PMC9075580

[bib10] Biderre-Petit C, Jézéquel D, Dugat-Bony E et al. Identification of microbial communities involved in the methane cycle of a freshwater meromictic lake: methane cycle in a stratified freshwater ecosystem. FEMS Microbiol Ecol. 2011;77:533–45. 10.1111/j.1574-6941.2011.01134.x. 21595728

[bib11] Blin K, Shaw S, Augustijn HE et al. antiSMASH 7.0: new and improved predictions for detection, regulation, chemical structures and visualisation. Nucleic Acids Res. 2023;51:W46–50. 10.1093/nar/gkad344. 37140036 PMC10320115

[bib12] Blum M, Andreeva A, Florentino LC et al. InterPro: the protein sequence classification resource in 2025. Nucleic Acids Res. 2025;53:D444–56. 10.1093/nar/gkae1082. 39565202 PMC11701551

[bib13] Bodelier PLE, Pérez G, Veraart AJ et al. Methanotroph ecology, environmental distribution and functioning. In: Lee EY (ed.), Methanotrophs. Vol 32. Cham: Springer International Publishing, 2019, 1–38.

[bib14] Bogachev AV, Murtazina RA, Skulachev VP. The Na^+^/e^−^ stoichiometry of the Na^+^-motive NADH : quinone oxidoreductase in *Vibrio alginolyticus*. FEBS Lett. 1997;409:475–7. 10.1016/S0014-5793(97)00536-X. 9224712

[bib15] Bowman J. The methanotrophs—the families *Methylococcaceae* and *Methylocystaceae*. In: Dworkin M, Falkow S, Rosenberg E, al. et (eds), The Prokaryotes. New York: Springer, 2006, 266–89. 10.1007/0-387-30745-1.

[bib16] Bowman JP, Sly LI, Nichols PD et al. Revised taxonomy of the methanotrophs: description of *Methylobacter* gen. nov., emendation of *Methylococcus*, validation of *Methylosinus* and *Methylocystis* species, and a proposal that the family *Methylococcaceae* includes only the group I methanotrophs. Int J Syst Bacteriol. 1993;43:735–53. 10.1099/00207713-43-4-735.

[bib18] Bowman JP. Methylosphaera. In: Trujillo ME, Dedysh S, DeVos P, al. et (eds), Bergey’s Manual of Systematics of Archaea and Bacteria. 1st edn. Hoboken: Wiley, 2015, 1–5.

[bib17] Bowman JP. The family *Methylococcaceae*. In: Rosenberg E, DeLong EF, Lory S et al. (eds), The Prokaryotes. Berlin, Heidelberg: Springer, 2014, 411–40. 10.1007/978-3-642-38922-1.

[bib20] Buchfink B, Reuter K, Drost H-G. Sensitive protein alignments at tree-of-life scale using DIAMOND. Nat Methods. 2021;18:366–8. 10.1038/s41592-021-01101-x. 33828273 PMC8026399

[bib21] Buchfink B, Xie C, Huson DH. Fast and sensitive protein alignment using DIAMOND. Nat Methods. 2015;12:59–60. 10.1038/nmeth.3176. 25402007

[bib22] Buck M, Garcia SL, Fernandez L et al. Comprehensive dataset of shotgun metagenomes from oxygen stratified freshwater lakes and ponds. Sci Data. 2021;8:131. 10.1038/s41597-021-00910-1. 33990618 PMC8121793

[bib23] Buckel W, Ermler U, Vonck J et al. The RNF/NQR redox pumps: a versatile system for energy transduction in bacteria and archaea. Appl Microbiol Biotechnol. 2025;109:148. 10.1007/s00253-025-13531-0. 40528048 PMC12174285

[bib24] Bussmann I, Rahalkar M, Schink B. Cultivation of methanotrophic bacteria in opposing gradients of methane and oxygen. FEMS Microbiol Ecol. 2006;56:331–44. 10.1111/j.1574-6941.2006.00076.x. 16689866

[bib25] Cabello-Yeves PJ, Picazo A, Roda-Garcia JJ et al. Vertical niche occupation and potential metabolic interplay of microbial consortia in a deeply stratified meromictic model lake. Limnol Oceanogr. 2023;68:2492–511. 10.1002/lno.12437.

[bib26] Cabrol L, Thalasso F, Gandois L et al. Anaerobic oxidation of methane and associated microbiome in anoxic water of Northwestern Siberian lakes. Sci Total Environ. 2020;736:139588. 10.1016/j.scitotenv.2020.139588. 32497884

[bib27] Camacho C, Coulouris G, Avagyan V et al. BLAST+: architecture and applications. BMC Bioinf. 2009;10:421. 10.1186/1471-2105-10-421. PMC2803857 20003500

[bib28] Caporaso GJ, Ackermann G, Apprill A et al. EMP 16S Illumina Amplicon Protocol V1. protocols.io, 2018. 10.17504/protocols.io.nuudeww

[bib29] Carere CR, McDonald B, Peach HA et al. Hydrogen oxidation influences glycogen accumulation in a verrucomicrobial methanotroph. Front Microbiol. 2019;10:1873. 10.3389/fmicb.2019.01873. 31474959 PMC6706786

[bib31] Chen KH-C, Wu H-H, Ke S-F et al. Bacteriohemerythrin bolsters the activity of the particulate methane monooxygenase (pMMO) in *Methylococcus capsulatus* (Bath). J Inorg Biochem. 2012;111:10–7. 10.1016/j.jinorgbio.2012.02.019. 22484247

[bib32] Chiriac M-C, Bulzu P-A, Andrei A-S et al. Ecogenomics sheds light on diverse lifestyle strategies in freshwater CPR. Microbiome. 2022;10:84. 10.1186/s40168-022-01274-3. 35659305 PMC9166423

[bib33] Chklovski A, Parks DH, Woodcroft BJ et al. CheckM2: a rapid, scalable and accurate tool for assessing microbial genome quality using machine learning. Nat Methods. 2023;20:1203–12. 10.1038/s41592-023-01940-w. 37500759

[bib34] Chu F, Lidstrom ME. XoxF acts as the predominant methanol dehydrogenase in the type I methanotroph *Methylomicrobium buryatense*. J Bacteriol. 2016;198:1317–25. 10.1128/JB.00959-15. 26858104 PMC4859581

[bib35] Chuckran PF, Flagg C, Propster J et al. Edaphic controls on genome size and GC content of bacteria in soil microbial communities. Soil Biol Biochem. 2023;178:108935. 10.1016/j.soilbio.2022.108935.

[bib36] Danilova OV, Suzina NE, Van De Kamp J et al. A new cell morphotype among methane oxidizers: a spiral-shaped obligately microaerophilic methanotroph from northern low-oxygen environments. ISME J. 2016;10:2734–43. 10.1038/ismej.2016.48. 27058508 PMC5113839

[bib38] de la Torre A, Metivier A, Chu F et al. Genome-scale metabolic reconstructions and theoretical investigation of methane conversion in *Methylomicrobium buryatense* strain 5G(B1). Microb Cell Fact. 2015;14:188. 10.1186/s12934-015-0377-3. 26607880 PMC4658805

[bib37] Dedysh SN, Knief C. Diversity and phylogeny of described aerobic methanotrophs. In: Kalyuzhnaya MG, Xing X-H (eds), Methane Biocatalysis: Paving the Way to Sustainability. Cham: Springer International Publishing, 2018, 17–42. 10.1007/978-3-319-74866-5.

[bib39] Deng Y, Liang C, Zhu X et al. *Methylomonadaceae* was the active and dominant methanotroph in Tibet lake sediments. ISME Commun. 2024;4:ycae032. 10.1093/ismeco/ycae032. 38524764 PMC10960969

[bib40] Ding H, Demple B. *In vivo* kinetics of a redox-regulated transcriptional switch. Proc Natl Acad Sci USA. 1997;94:8445–9. 10.1073/pnas.94.16.8445. 9237996 PMC22951

[bib41] Eddy SR Accelerated profile HMM searches. PLoS Comput Biol. 2011;7:e1002195. 10.1371/journal.pcbi.1002195. 22039361 PMC3197634

[bib42] Edgar RC. MUSCLE: multiple sequence alignment with high accuracy and high throughput. Nucleic Acids Res. 2004;32:1792–7. 10.1093/nar/gkh340. 15034147 PMC390337

[bib43] Eren AM, Kiefl E, Shaiber A et al. Community-led, integrated, reproducible multi-omics with anvi’o. Nat Microbiol. 2021;6:3–6. 10.1038/s41564-020-00834-3. 33349678 PMC8116326

[bib44] Etminan M, Myhre G, Highwood EJ et al. Radiative forcing of carbon dioxide, methane, and nitrous oxide: a significant revision of the methane radiative forcing. Geophys Res Lett. 2016;43:12 614–23. 10.1002/2016GL071930.

[bib45] Flynn JD, Hirayama H, Sakai Y et al. Draft genome sequences of gammaproteobacterial methanotrophs isolated from marine ecosystems. Genome Announc. 2016;4:e01629–15. 10.1128/genomeA.01629-15. 26798114 PMC4722281

[bib46] Foerstner KU, Von Mering C, Hooper SD et al. Environments shape the nucleotide composition of genomes. EMBO Rep. 2005;6:1208–13. 10.1038/sj.embor.7400538. 16200051 PMC1369203

[bib47] Gafni A, Rubin-Blum M, Murrell C et al. Survival strategies of aerobic methanotrophs under hypoxia in methanogenic lake sediments. Environ Microbiome. 2024;19:44. 10.1186/s40793-024-00586-1. 38956741 PMC11218250

[bib48] Garber AI, Nealson KH, Okamoto A et al. FeGenie: a comprehensive tool for the identification of iron genes and iron gene neighborhoods in genome and metagenome assemblies. Front Microbiol. 2020;11:37. 10.3389/fmicb.2020.00037. 32082281 PMC7005843

[bib49] Giuffrè A, Borisov VB, Arese M et al. Cytochrome *bd* oxidase and bacterial tolerance to oxidative and nitrosative stress. Biochim Biophys Acta BBA Bioenerg. 2014;1837:1178–87. 10.1016/j.bbabio.2014.01.016. 24486503

[bib50] Goris J, Konstantinidis KT, Klappenbach JA et al. DNA–DNA hybridization values and their relationship to whole-genome sequence similarities. Int J Syst Evol Microbiol. 2007;57:81–91. 10.1099/ijs.0.64483-0. 17220447

[bib51] Graef C, Hestnes AG, Svenning MM et al. The active methanotrophic community in a wetland from the High Arctic: methanotrophs in the High Arctic. Environ Microbiol Rep. 2011;3:466–72. 10.1111/j.1758-2229.2010.00237.x. 23761309

[bib52] Graf JS, Mayr MJ, Marchant HK et al. Bloom of a denitrifying methanotroph, ‘*Candidatus* Methylomirabilis limnetica’, in a deep stratified lake. Environ Microbiol. 2018;20:2598–614. 10.1111/1462-2920.14285. 29806730

[bib53] Greening C, Biswas A, Carere CR et al. Genomic and metagenomic surveys of hydrogenase distribution indicate H_2_ is a widely utilised energy source for microbial growth and survival. ISME J. 2016;10:761–77. 10.1038/ismej.2015.153. 26405831 PMC4817680

[bib54] Grégoire DS, George NA, Hug LA. Microbial methane cycling in a landfill on a decadal time scale. Nat Commun. 2023;14:7402. 10.1038/s41467-023-43129-x. 37973978 PMC10654671

[bib55] Guilloton M, Karst F. Isolation and characterization of *Escherichia coli* mutants lacking inducible cyanase. J Gen Microbiol. 1987;133:645–53. 3309165 10.1099/00221287-133-3-645

[bib56] Hackl T, Ankenbrand M, van Adrichem B et al. gggenomes: effective and versatile visualizations for comparative genomics. arXiv 2024. 10.48550/ARXIV.2411.13556.

[bib57] Hakobyan A, Liesack W. Unexpected metabolic versatility among type II methanotrophs in the *Alphaproteobacteria*. Biol Chem. 2020;401:1469–77. 10.1515/hsz-2020-0200. 32769217

[bib58] Hakobyan A, Zhu J, Glatter T et al. Hydrogen utilization by *Methylocystis* sp. strain SC2 expands the known metabolic versatility of type IIa methanotrophs. Metab Eng. 2020;61:181–96. 10.1016/j.ymben.2020.05.003. 32479801

[bib59] Hamilton R, Kits KD, Ramonovskaya VA et al. Draft genomes of gammaproteobacterial methanotrophs isolated from terrestrial ecosystems. Genome Announc. 2015;3:e00515–15. 10.1128/genomeA.00515-15. 26044417 PMC4457054

[bib60] Han D, Dedysh SN, Liesack W. Unusual genomic traits suggest *Methylocystis bryophila* S285 to be well adapted for life in peatlands. Genome Biol Evol. 2018;10:623–8. 10.1093/gbe/evy025. 29390143 PMC5808792

[bib61] Hanson RS, Hanson TE. Methanotrophic bacteria. Microbiol Rev. 1996;60:439–71. 10.1128/mr.60.2.439-471.1996. 8801441 PMC239451

[bib62] Hao Q, Wang O, Jiao J-Y et al. *Methylobacter* couples methane oxidation and N_2_O production in hypoxic wetland soil. Soil Biol Biochem. 2022;175:108863. 10.1016/j.soilbio.2022.108863.

[bib63] He R, Wang J, Pohlman JW et al. Metabolic flexibility of aerobic methanotrophs under anoxic conditions in Arctic lake sediments. ISME J. 2022;16:78–90. 10.1038/s41396-021-01049-y. 34244610 PMC8692461

[bib64] Hernandez ME, Beck DAC, Lidstrom ME et al. Oxygen availability is a major factor in determining the composition of microbial communities involved in methane oxidation. PeerJ. 2015;3:e801. 10.7717/peerj.801. 25755930 PMC4349146

[bib65] Hoang DT, Chernomor O, Von Haeseler A et al. UFBoot2: improving the ultrafast bootstrap approximation. Mol Biol Evol. 2018;35:518–22. 10.1093/molbev/msx281. 29077904 PMC5850222

[bib66] Hogendoorn C, Picone N, Van Hout F et al. Draft genome of a novel methanotrophic *Methylobacter* sp. from the volcanic soils of Pantelleria Island. Antonie Van Leeuwenhoek. 2021;114:313–24. 10.1007/s10482-021-01525-7. 33566237 PMC7902576

[bib67] Hreha TN, Foreman S, Duran-Pinedo A et al. The three NADH dehydrogenases of *Pseudomonas aeruginosa*: their roles in energy metabolism and links to virulence. PLoS One. 2021;16:e0244142. 10.1371/journal.pone.0244142. 33534802 PMC7857637

[bib68] Hu E-Z, Lan X-R, Liu Z-L et al. A positive correlation between GC content and growth temperature in prokaryotes. BMC Genomics. 2022;23:110. 10.1186/s12864-022-08353-7. 35139824 PMC8827189

[bib69] Hyatt D, Chen G-L, LoCascio PF et al. Prodigal: prokaryotic gene recognition and translation initiation site identification. BMC Bioinf. 2010;11:119. 10.1186/1471-2105-11-119. PMC2848648 20211023

[bib70] Iguchi H, Yurimoto H, Sakai Y. Soluble and particulate methane monooxygenase gene clusters of the type I methanotroph *Methylovulum miyakonense* HT12: sMMO and pMMO gene clusters of *Methylovulum miyakonense* HT12. FEMS Microbiol Lett. 2010;312:71–6. 10.1111/j.1574-6968.2010.02101.x. 20846142

[bib71] Islam MM, Le T, Daggumati SR et al. Investigation of microbial community interactions between Lake Washington methanotrophs using genome-scale metabolic modeling. PeerJ. 2020;8:e9464. 10.7717/peerj.9464. 32655999 PMC7333651

[bib72] Ito T, Gallegos R, Matano LM et al. Genetic and biochemical analysis of anaerobic respiration in *Bacteroides fragilis* and its importance *in vivo*. mBio. 2020;11. 10.1128/mbio.03238-19. PMC7002350 32019804

[bib73] Jackson RB, Saunois M, Bousquet P et al. Increasing anthropogenic methane emissions arise equally from agricultural and fossil fuel sources. Environ Res Lett. 2020;15:071002. 10.1088/1748-9326/ab9ed2.

[bib74] Jaffe AL, Bardot C, Le Jeune A-H et al. Variable impact of geochemical gradients on the functional potential of bacteria, archaea, and phages from the permanently stratified Lac Pavin. Microbiome. 2023;11:14. 10.1186/s40168-022-01416-7. 36694212 PMC9875498

[bib75] Jain C, Rodriguez-R LM, Phillippy AM et al. High throughput ANI analysis of 90 K prokaryotic genomes reveals clear species boundaries. Nat Commun. 2018;9:5114. 10.1038/s41467-018-07641-9. 30504855 PMC6269478

[bib76] Kaila VRI, Wikström M. Architecture of bacterial respiratory chains. Nat Rev Microbiol. 2021;19:319–30. 10.1038/s41579-020-00486-4. 33437024

[bib77] Kallistova A, Oshkin I, Rusanov I et al. Profile distribution of methane oxidation in reduced lake sediments suggests utilization of NO-dismutation pathway by aerobic methanotrophic bacteria. Microbiology. 2024;94:831–51. 10.2139/ssrn.4805437.

[bib78] Kalyaanamoorthy S, Minh BQ, Wong TKF et al. ModelFinder: fast model selection for accurate phylogenetic estimates. Nat Methods. 2017;14:587–9. 10.1038/nmeth.4285. 28481363 PMC5453245

[bib79] Kalyuzhnaya MG, Lamb AE, McTaggart TL et al. Draft genome sequences of gammaproteobacterial methanotrophs isolated from Lake Washington sediment. Genome Announc. 2015;3:e00103–15. 10.1128/genomeA.00103-15. 25767239 PMC4357761

[bib80] Kalyuzhnaya MG, Yang S, Rozova ON et al. Highly efficient methane biocatalysis revealed in a methanotrophic bacterium. Nat Commun. 2013;4:2785. 10.1038/ncomms3785. 24302011

[bib81] Kao W-C, Wang VC-C, Huang Y-C et al. Isolation, purification and characterization of hemerythrin from *Methylococcus capsulatus* (Bath). J Inorg Biochem. 2008;102:1607–14. 10.1016/j.jinorgbio.2008.02.008. 18397812

[bib82] Katoh K, Standley DM. MAFFT multiple sequence alignment software version 7: improvements in performance and usability. Mol Biol Evol. 2013;30:772–80. 10.1093/molbev/mst010. 23329690 PMC3603318

[bib83] Keltjens JT, Pol A, Reimann J et al. PQQ-dependent methanol dehydrogenases: rare-earth elements make a difference. Appl Microbiol Biotechnol. 2014;98:6163–83. 10.1007/s00253-014-5766-8. 24816778

[bib84] Khanongnuch R, Mangayil R, Svenning MM et al. Characterization and genome analysis of a psychrophilic methanotroph representing a ubiquitous *Methylobacter* spp. cluster in boreal lake ecosystems. ISME Commun. 2022;2:85. 10.1038/s43705-022-00172-x. 37938755 PMC9723741

[bib85] Khatri K, Mohite J, Pandit P et al. Isolation, description and genome analysis of a putative novel *Methylobacter* species (‘*Ca*. Methylobacter coli’) isolated from the faeces of a blackbuck (Indian antelope). Microbiol Res. 2021;12:513–23. 10.3390/microbiolres12020035.

[bib86] Khatri K, Mohite JA, Pandit PS et al. Description of ‘*Ca*. Methylobacter oryzae’ KRF1, a novel species from the environmentally important *methylobacter* clade 2. Antonie Van Leeuwenhoek. 2020;113:729–35. 10.1007/s10482-019-01369-2. 31813064

[bib87] Kim D, Park S, Chun J. Introducing EzAAI: a pipeline for high throughput calculations of prokaryotic average amino acid identity. J Microbiol. 2021;59:476–80. 10.1007/s12275-021-1154-0. 33907973

[bib88] Kim M, Oh H-S, Park S-C et al. Towards a taxonomic coherence between average nucleotide identity and 16S rRNA gene sequence similarity for species demarcation of prokaryotes. Int J Syst Evol Microbiol. 2014;64:346–51. 10.1099/ijs.0.059774-0. 24505072

[bib89] King GM, Roslev P, Skovgaard H. Distribution and rate of methane oxidation in sediments of the Florida Everglades. Appl Environ Microbiol. 1990;56:2902–11. 10.1128/aem.56.9.2902-2911.1990. 16348299 PMC184862

[bib90] King GM. Regulation by light of methane emissions from a wetland. Nature. 1990;345:513–5. 10.1038/345513a0.

[bib91] Kits KD, Klotz MG, Stein LY. Methane oxidation coupled to nitrate reduction under hypoxia by the gammaproteobacterium *Methylomonas denitrificans* , sp. nov. type strain FJG1. Environ Microbiol. 2015;17:3219–32. 10.1111/1462-2920.12772. 25580993

[bib92] Knief C. Diversity and habitat preferences of cultivated and uncultivated aerobic methanotrophic bacteria evaluated based on *pmoA* as molecular marker. Front Microbiol. 2015;6. 10.3389/fmicb.2015.01346. PMC4678205 26696968

[bib93] Konstantinidis KT, Rosselló-Móra R, Amann R. Uncultivated microbes in need of their own taxonomy. ISME J. 2017;11:2399–406. 10.1038/ismej.2017.113. 28731467 PMC5649169

[bib94] Koo M, Lee J, Rah S et al. A reducing system of the superoxide sensor SoxR in *Escherichia coli*. EMBO J. 2003;22:2614–22. 10.1093/emboj/cdg252. 12773378 PMC156749

[bib95] Krause SMB, Van Den Berg NI, Brenzinger K et al. Beyond methane consumption: exploring the potential of methanotrophic bacteria to produce secondary metabolites. ISME Commun. 2025;5:ycaf030. 10.1093/ismeco/ycaf030. 40177465 PMC11964084

[bib96] Lan X, Thoning KW, Dlugokencky EJ et al. Trends in globally-averaged CH_4_, N_2_O, and SF_6_. Version 2025-09, Barrow: NOAA Global Monitoring Laboratory, 2022. 10.15138/P8XG-AA10.

[bib97] Leone V, Pogoryelov D, Meier T et al. On the principle of ion selectivity in Na^+^/H^+^-coupled membrane proteins: experimental and theoretical studies of an ATP synthase rotor. Proc Natl Acad Sci USA. 2015;112. 10.1073/pnas.1421202112. PMC4364180 25713346

[bib98] Letunic I, Bork P. Interactive Tree of Life (iTOL) v6: recent updates to the phylogenetic tree display and annotation tool. Nucleic Acids Res. 2024;52:W78–82. 10.1093/nar/gkae268. 38613393 PMC11223838

[bib99] Leung PM, Greening C. Greening lab metabolic marker gene databases. Monash University. Collection. 2020. 10.26180/c.5230745,

[bib100] Li B, Mao Z, Xue J et al. Metabolic versatility of aerobic methane-oxidizing bacteria under anoxia in aquatic ecosystems. Environ Microbiol Rep. 2024;16:e70002. 10.1111/1758-2229.70002. 39232853 PMC11374530

[bib101] Li B, Tao Y, Mao Z et al. Iron oxides act as an alternative electron acceptor for aerobic methanotrophs in anoxic lake sediments. Water Res. 2023;234:119833. 10.1016/j.watres.2023.119833. 36889095

[bib102] Li R, Yuan Y, Xi B et al. Anaerobic methane oxidation coupled with denitrification mitigates soil nitrous oxide emissions. Sci Adv. 2025;11. 10.1126/sciadv.adv1410. PMC12154179 40498835

[bib103] Lidstrom ME. Isolation and characterization of marine methanotrophs. Antonie Van Leeuwenhoek. 1988;54:189–99. 10.1007/BF00443577. 3138946

[bib104] Liu C, Schmitz RA, Pol A et al. Active coexistence of the novel gammaproteobacterial methanotroph ‘*Ca*. Methylocalor cossyra’ CH1 and verrucomicrobial methanotrophs in acidic, hot geothermal soil. Environ Microbiol. 2024;26:e16602. 10.1111/1462-2920.16602. 38454738

[bib105] Ma B, Lu C, Wang Y et al. A genomic catalogue of soil microbiomes boosts mining of biodiversity and genetic resources. Nat Commun. 2023;14:7318. 10.1038/s41467-023-43000-z. 37951952 PMC10640626

[bib106] Magnuson E, Altshuler I, Fernández-Martínez MÁ et al. Active lithoautotrophic and methane-oxidizing microbial community in an anoxic, sub-zero, and hypersaline High Arctic spring. ISME J. 2022;16:1798–808. 10.1038/s41396-022-01233-8. 35396347 PMC9213412

[bib107] Martinez-Cruz K, Leewis M-C, Herriott IC et al. Anaerobic oxidation of methane by aerobic methanotrophs in sub-arctic lake sediments. Sci Total Environ. 2017;607–608:23–31. 10.1016/j.scitotenv.2017.06.187. 28686892

[bib108] Mayr MJ, Zimmermann M, Guggenheim C et al. Niche partitioning of methane-oxidizing bacteria along the oxygen–methane counter gradient of stratified lakes. ISME J. 2020;14:274–87. 10.1038/s41396-019-0515-8. 31624343 PMC6908591

[bib109] Meier-Kolthoff JP, Auch AF, Klenk H-P et al. Genome sequence-based species delimitation with confidence intervals and improved distance functions. BMC Bioinf. 2013;14:60. 10.1186/1471-2105-14-60. PMC3665452 23432962

[bib110] Meier-Kolthoff JP, Carbasse JS, Peinado-Olarte RL et al. TYGS and LPSN: a database tandem for fast and reliable genome-based classification and nomenclature of prokaryotes. Nucleic Acids Res. 2022;50:D801–7. 10.1093/nar/gkab902. 34634793 PMC8728197

[bib111] Meier-Kolthoff JP, Göker M. TYGS is an automated high-throughput platform for state-of-the-art genome-based taxonomy. Nat Commun. 2019;10:2182. 10.1038/s41467-019-10210-3. 31097708 PMC6522516

[bib112] Meier-Kolthoff JP, Hahnke RL, Petersen J et al. Complete genome sequence of DSM 30083T, the type strain (U5/41T) of *Escherichia coli*, and a proposal for delineating subspecies in microbial taxonomy. Stand Genomic Sci. 2014;9:2. 10.1186/1944-3277-9-2. 25780495 PMC4334874

[bib113] Meier-Kolthoff JP, Klenk H-P, Göker M. Taxonomic use of DNA G+C content and DNA–DNA hybridization in the genomic age. Int J Syst Evol Microbiol. 2014;64:352–6. 10.1099/ijs.0.056994-0. 24505073

[bib114] Michaud AB, Dore JE, Achberger AM et al. Microbial oxidation as a methane sink beneath the West Antarctic Ice Sheet. Nat Geosci. 2017;10:582–6. 10.1038/ngeo2992.

[bib115] Minh BQ, Schmidt HA, Chernomor O et al. IQ-TREE 2: new models and efficient methods for phylogenetic inference in the genomic era. Mol Biol Evol. 2020;37:1530–4. 10.1093/molbev/msaa015. 32011700 PMC7182206

[bib116] Myhre G, Shindell D, Bréon F et al. Anthropogenic and natural radiative forcing, climate change 2013: the physical science basis. In: Stocker T.F., Qin D., Plattner G.-K., et al. (eds), Contribution of Working group I to the Fifth Assessment Report of the Intergovernmental Panel on Climate Change, Cambridge University Press, 2013, 659–740. 10.1017/CBO9781107415324.018

[bib117] Nariya S, Kalyuzhnaya MG. Hemerythrins enhance aerobic respiration in *Methylomicrobium alcaliphilum* 20ZR, a methane-consuming bacterium. FEMS Microbiol Lett. 2020;367:fnaa003. 10.1093/femsle/fnaa003. 32053143

[bib118] Nercessian O, Noyes E, Kalyuzhnaya MG et al. Bacterial populations active in metabolism of C_1_ compounds in the fediment of Lake Washington, a freshwater lake. Appl Environ Microbiol. 2005;71:6885–99. 10.1128/AEM.71.11.6885-6899.2005. 16269723 PMC1287692

[bib119] Nguyen L-T, Schmidt HA, Von Haeseler A et al. IQ-TREE: a fast and effective stochastic algorithm for estimating maximum-likelihood phylogenies. Mol Biol Evol. 2015;32:268–74. 10.1093/molbev/msu300. 25371430 PMC4271533

[bib120] Nguyen N-L, Yu W-J, Gwak J-H et al. Genomic insights into the acid adaptation of novel methanotrophs enriched from acidic forest soils. Front Microbiol. 2018;9:1982. 10.3389/fmicb.2018.01982. 30210468 PMC6119699

[bib121] Nguyen N-L, Yu W-J, Yang H-Y et al. A novel methanotroph in the genus *Methylomonas* that contains a distinct clade of soluble methane monooxygenase. J Microbiol. 2017;55:775–82. 10.1007/s12275-017-7317-3. 28956349

[bib122] Nweze JA, Tláskal V, Wutkowska M et al. Regulators of aerobic and anaerobic methane oxidation in two pristine temperate peatland types. FEMS Microbiol Ecol. 2024;100:fiae153. 10.1093/femsec/fiae153. 39510969 PMC11585280

[bib123] Omelchenko M, Vasilyeva L, Zavarzin G et al. A novel psychrophilic methanotroph of the genus *Methylobacter*. Микробиология. 1996;65:384–9.

[bib124] Omelchenko M, Vasilyeva V, Zavarzin G. Psychrophilic methanotroph from tundra soil. Curr Microbiol. 1993;27:255–9. 10.1007/BF01575988.

[bib125] Orata FD, Meier-Kolthoff JP, Sauvageau D et al. Phylogenomic analysis of the gammaproteobacterial methanotrophs (order *Methylococcales*) calls for the reclassification of members at the genus and species levels. Front Microbiol. 2018;9:3162. 10.3389/fmicb.2018.03162. 30631317 PMC6315193

[bib126] Oremland RS, Culbertson CW. Importance of methane-oxidizing bacteria in the methane budget as revealed by the use of a specific inhibitor. Nature. 1992;356:421–3. 10.1038/356421a0.

[bib127] Osborne CD, Haritos VS. Horizontal gene transfer of three co-inherited methane monooxygenase systems gave rise to methanotrophy in the *Proteobacteria*. Mol Phylogenet Evol. 2018;129:171–81. 10.1016/j.ympev.2018.08.010. 30149053

[bib128] Oshkin IY, Beck DA, Lamb AE et al. Methane-fed microbial microcosms show differential community dynamics and pinpoint taxa involved in communal response. ISME J. 2015;9:1119–29. 10.1038/ismej.2014.203. 25333464 PMC4409156

[bib129] Oshkin IY, Miroshnikov KK, Grouzdev DS et al. Pan-genome-based analysis as a framework for demarcating two closely related methanotroph genera *Methylocystis* and *Methylosinus*. Microorganisms. 2020;8:768. 10.3390/microorganisms8050768. 32443820 PMC7285482

[bib130] Parks DH, Chaumeil P-A, Mussig AJ et al. GTDB release 10: a complete and systematic taxonomy for 715 230 bacterial and 17 245 archaeal genomes. Nucleic Acids Res. 2025;53:gkaf1040. 10.1093/nar/gkaf1040. PMC12807784 41123020

[bib131] Parks DH, Rinke C, Chuvochina M et al. Recovery of nearly 8,000 metagenome-assembled genomes substantially expands the tree of life. Nat Microbiol. 2017;2:1533–42. 10.1038/s41564-017-0012-7. 28894102

[bib132] Patil SK, Islam T, Tveit A et al. Targeting methanotrophs and isolation of a novel psychrophilic *Methylobacter* species from a terrestrial Arctic alkaline methane seep in Lagoon Pingo, Central Spitsbergen (78° N). Antonie Van Leeuwenhoek. 2024;117:60. 10.1007/s10482-024-01953-1. 38517574 PMC10959801

[bib133] Pedron R, Esposito A, Bianconi I et al. Genomic and metagenomic insights into the microbial community of a thermal spring. Microbiome. 2019;7:8. 10.1186/s40168-019-0625-6. 30674352 PMC6343286

[bib134] Pfeifer F. Distribution, formation and regulation of gas vesicles. Nat Rev Microbiol. 2012;10:705–15. 10.1038/nrmicro2834. 22941504

[bib135] Potužák J, Hůda J, Pechar L. Changes in fish production effectivity in eutrophic fishponds—impact of zooplankton structure. Aquac Int. 2007;15:201–10.

[bib136] Pritchard L, Glover RH, Humphris S et al. Genomics and taxonomy in diagnostics for food security: soft-rotting enterobacterial plant pathogens. Anal Methods. 2016;8:12–24. 10.1039/C5AY02550H.

[bib137] Puri AW, Mevers E, Ramadhar TR et al. Tundrenone: an atypical secondary metabolite from bacteria with highly restricted primary metabolism. J Am Chem Soc. 2018;140:2002–6. 10.1021/jacs.7b12240. 29361220 PMC5817624

[bib138] R Core Team . R: a language and environment for statistical computing. Vienna: R Foundation for Statistical Computing, 2024.

[bib139] Raba DA, Rosas-Lemus M, Menzer WM et al. Characterization of the *Pseudomonas aeruginosa* NQR complex, a bacterial proton pump with roles in autopoisoning resistance. J Biol Chem. 2018;293:15664–77. 10.1074/jbc.RA118.003194. 30135204 PMC6177581

[bib140] Rahalkar MC, Bahulikar RA. Hemerythrins are widespread and conserved for methanotrophic guilds. Gene Rep. 2018;11:250–4. 10.1016/j.genrep.2018.04.008.

[bib141] Reim A, Lüke C, Krause S et al. One millimetre makes the difference: high-resolution analysis of methane-oxidizing bacteria and their specific activity at the oxic–anoxic interface in a flooded paddy soil. ISME J. 2012;6:2128–39. 10.1038/ismej.2012.57. 22695859 PMC3475382

[bib142] Reis PCJ, Tsuji JM, Weiblen C et al. Enigmatic persistence of aerobic methanotrophs in oxygen-limiting freshwater habitats. ISME J. 2024;18:wrae041. 10.1093/ismejo/wrae041. 38470309 PMC11008690

[bib143] Reshetnikov AS, Khmelenina VN, Mustakhimov II et al. Diversity and phylogeny of the ectoine biosynthesis genes in aerobic, moderately halophilic methylotrophic bacteria. Extremophiles. 2011;15:653–63. 10.1007/s00792-011-0396-x. 21971967

[bib144] Rissanen A, Saarenheimo J, Tiirola M et al. Gammaproteobacterial methanotrophs dominate methanotrophy in aerobic and anaerobic layers of boreal lake waters. Aquat Microb Ecol. 2018;81:257–76. 10.3354/ame01874.

[bib203_703_051626] Rissanen AJ, Hestnes AG, Khanongnuch R et al. *Methylobacter arcticus* sp. nov. isolated from a coal mine biofilm in the high Arctic Svalbard. International Journal of Systematic and Evolutionary Microbiology. 2025b;75:006984. 10.1099/ijsem.0.006984 41313628 PMC12662621

[bib145] Rissanen AJ, Mangayil R, Svenning MM et al. Draft genome sequence data of a psychrophilic tundra soil methanotroph, *Methylobacter psychrophilus* Z-0021 (DSM 9914). Data Brief. 2022;45:108689. 10.1016/j.dib.2022.108689. 36426084 PMC9679665

[bib146] Rissanen AJ, Mangayil R, Tveit AT et al. Dissolved organic matter and sulfide enhance the CH_4_ consumption of a psychrophilic lake methanotroph, *Methylobacter* sp. S3L5C. Microbiol Spectr. 2025a;13. 10.1128/spectrum.03133-24. PMC12211021 40488469

[bib147] Rissanen AJ, Saarela T, Jäntti H et al. Vertical stratification patterns of methanotrophs and their genetic controllers in water columns of oxygen-stratified boreal lakes. FEMS Microbiol Ecol. 2021;97:fiaa252. 10.1093/femsec/fiaa252. 33316049 PMC7840105

[bib148] Rocha U, Coelho Kasmanas J, Kallies R et al. MuDoGeR : multi-domain genome recovery from metagenomes made easy. Mol Ecol Resour. 2024;24:e13904. 10.1111/1755-0998.13904. 37994269

[bib149] Rodrigues JFM, Tackmann J, Malfertheiner L et al. The MicrobeAtlas database: global trends and insights into Earth’s microbial ecosystems. bioRxiv. 2025. 10.1101/2025.07.18.665519. 41747730

[bib150] Roldán DM, Menes RJ. Characterisation of ‘*Candidatus* Methylobacter titanis’ sp. nov., a putative novel species of *Methylobacter* clade 2 and their distribution in sediments of freshwater lakes in maritime Antarctica. Antonie Van Leeuwenhoek. 2023;116:721–38. 10.1007/s10482-023-01840-1. 37227602

[bib151] Ruff SE, Humez P, De Angelis IH et al. Hydrogen and dark oxygen drive microbial productivity in diverse groundwater ecosystems. Nat Commun. 2023;14:3194. 10.1038/s41467-023-38523-4. 37311764 PMC10264387

[bib152] Ruff SE, Schwab L, Vidal E et al. Widespread occurrence of dissolved oxygen anomalies, aerobic microbes, and oxygen-producing metabolic pathways in apparently anoxic environments. FEMS Microbiol Ecol. 2024;100:fiae132. 10.1093/femsec/fiae132. 39327011 PMC11549561

[bib153] Sapart CJ, Monteil G, Prokopiou M et al. Natural and anthropogenic variations in methane sources during the past two millennia. Nature. 2012;490:85–8. 10.1038/nature11461. 23038470

[bib154] Savvichev A, Rusanov I, Dvornikov Y et al. The water column of the Yamal tundra lakes as a microbial filter preventing methane emission. Biogeosciences. 2021;18:2791–807. 10.5194/bg-18-2791-2021.

[bib155] Schöner TA, Gassel S, Osawa A et al. Aryl polyenes, a highly abundant class of bacterial natural products, are functionally related to antioxidative carotenoids. ChemBioChem. 2016;17:247–53. 26629877 10.1002/cbic.201500474

[bib156] Schorn S, Graf JS, Littmann S et al. Persistent activity of aerobic methane-oxidizing bacteria in anoxic lake waters due to metabolic versatility. Nat Commun. 2024;15:5293. 10.1038/s41467-024-49602-5. 38906896 PMC11192741

[bib157] Schulz S, Iglesias-Cans M, Krah A et al. A new type of Na^+^-driven ATP synthase membrane rotor with a two-carboxylate ion-coupling motif. PLoS Biol. 2013;11:e1001596. 10.1371/journal.pbio.1001596. 23824040 PMC3692424

[bib158] Scott DR, Weeks D, Hong C et al. The role of internal urease in acid resistance of *Helicobacter pylori*. Gastroenterology. 1998;114:58–70. 10.1016/S0016-5085(98)70633-X. 9428219

[bib159] Semrau JD. Bioremediation via methanotrophy: overview of recent findings and suggestions for future research. Front Microbiol. 2011;2. 10.3389/fmicb.2011.00209. PMC3191459 22016748

[bib160] Seppey CVW, Cabrol L, Thalasso F et al. Biogeography of microbial communities in high-latitude ecosystems: contrasting drivers for methanogens, methanotrophs and global prokaryotes. Environ Microbiol. 2023;25:3364–86. 10.1111/1462-2920.16526. 37897125

[bib161] Shaffer M, Borton MA, McGivern BB et al. DRAM for distilling microbial metabolism to automate the curation of microbiome function. Nucleic Acids Res. 2020;48:8883–900. 10.1093/nar/gkaa621. 32766782 PMC7498326

[bib162] Singleton CM, McCalley CK, Woodcroft BJ et al. Methanotrophy across a natural permafrost thaw environment. ISME J. 2018;12:2544–58. 10.1038/s41396-018-0065-5. 29955139 PMC6155033

[bib163] Slobodkin AI, Ratnikova NM, Slobodkina GB et al. Composition and metabolic potential of Fe(III)-reducing enrichment cultures of methanotrophic ANME-2a archaea and associated bacteria. Microorganisms. 2023;11:555. 10.3390/microorganisms11030555. 36985129 PMC10052568

[bib164] Smith GJ, Angle JC, Solden LM et al. Members of the genus *Methylobacter* are inferred to account for the majority of aerobic methane oxidation in oxic soils from a freshwater wetland. mBio. 2018;9:e00815–18. 10.1128/mBio.00815-18. 30401770 PMC6222125

[bib165] Smith KS, Costello AM, Lidstrom ME. Methane and trichloroethylene oxidation by an estuarine methanotroph, *Methylobacter* sp. strain BB5.1. Appl Environ Microbiol. 1997;63:4617–20. 10.1128/aem.63.11.4617-4620.1997. 9361449 PMC168782

[bib166] Søndergaard D, Pedersen CNS, Greening C. HydDB: a web tool for hydrogenase classification and analysis. Sci Rep. 2016;6:34212. 10.1038/srep34212. 27670643 PMC5037454

[bib167] Spero MA, Aylward FO, Currie CR et al. Phylogenomic analysis and predicted physiological role of the proton-translocating NADH:quinone oxidoreductase (complex I) across bacteria. mBio. 2015;6. 10.1128/mbio.00389-15. PMC4453560 25873378

[bib168] Stanley SH, Dalton H. Role of ribulose-1,5-bisphosphate carboxylase/oxygenase in *Methylococcus capsulatus* (Bath). Microbiology. 1982;128:2927–35. 10.1099/00221287-128-12-2927.

[bib169] Steinegger M, Söding J. MMseqs2 enables sensitive protein sequence searching for the analysis of massive data sets. Nat Biotechnol. 2017;35:1026–8. 10.1038/nbt.3988. 29035372

[bib170] Stingl K, Altendorf K, Bakker EP. Acid survival of *Helicobacter pylori*: how does urease activity trigger cytoplasmic pH homeostasis?. Trends Microbiol. 2002;10:70–4. 10.1016/S0966-842X(01)02287-9. 11827807

[bib171] Sullivan JP, Dickinson D, Chase HA. Methanotrophs, *Methylosinus trichosporium* OB3b, sMMO, and their application to bioremediation. Crit Rev Microbiol. 1998;24:335–73. 10.1080/10408419891294217. 9887367

[bib172] Svenning MM, Hestnes AG, Wartiainen I et al. Genome sequence of the Arctic methanotroph *Methylobacter tundripaludum* SV96. J Bacteriol. 2011;193:6418–9. 10.1128/JB.05380-11. 21725021 PMC3209220

[bib173] Tavormina PL, Orphan VJ, Kalyuzhnaya MG et al. A novel family of functional operons encoding methane/ammonia monooxygenase-related proteins in gammaproteobacterial methanotrophs. Environ Microbiol Rep. 2011;3:91–100. 10.1111/j.1758-2229.2010.00192.x. 23761236

[bib6] The UniProt Consortium, Bateman A, Martin M-J et al. UniProt: the universal protein knowledgebase in 2025. Nucleic Acids Res. 2025;53:D609–17. 39552041 10.1093/nar/gkae1010 PMC11701636

[bib174] Trotsenko YA, Khmelenina VN. Aerobic methanotrophic bacteria of cold ecosystems. FEMS Microbiol Ecol. 2005;53:15–26. 10.1016/j.femsec.2005.02.010. 16329925

[bib175] Tveit A, Schwacke R, Svenning MM et al. Organic carbon transformations in high-Arctic peat soils: key functions and microorganisms. ISME J. 2013;7:299–311. 10.1038/ismej.2012.99. 22955232 PMC3554415

[bib176] Tveit AT, Söllinger A, Rainer EM et al. Thermal acclimation of methanotrophs from the genus *Methylobacter*. ISME J. 2023;17:1–11. 36650275 10.1038/s41396-023-01363-7 PMC10030640

[bib177] van Grinsven S, Sinninghe Damsté JS, Abdala Asbun A et al. Methane oxidation in anoxic lake water stimulated by nitrate and sulfate addition. Environ Microbiol. 2020a;22:766–82. 10.1111/1462-2920.14886. 31814267 PMC7027835

[bib178] van Grinsven S, Sinninghe Damsté JS, Harrison J et al. Impact of electron acceptor availability on methane-influenced microorganisms in an enrichment culture obtained from as stratified lake. Front Microbiol. 2020b;11:715. 10.3389/fmicb.2020.00715. 32477281 PMC7240106

[bib179] Van Keulen G, Hopwood DA, Dijkhuizen L et al. Gas vesicles in actinomycetes: old buoys in novel habitats?. Trends Microbiol. 2005;13:350–4. 10.1016/j.tim.2005.06.006. 15993071

[bib180] Veraart AJ, Garbeva P, van Beersum F et al. Living apart together—bacterial volatiles influence methanotrophic growth and activity. ISME J. 2018;12:1163–6. 10.1038/s41396-018-0055-7. 29382947 PMC5864204

[bib181] Vigderovich H, Eckert W, Elvert M et al. Aerobic methanotrophy increases the net iron reduction in methanogenic lake sediments. Front Microbiol. 2023;14:1206414. 10.3389/fmicb.2023.1206414. 37577416 PMC10415106

[bib182] Vignais PM, Billoud B, Meyer J. Classification and phylogeny of hydrogenases. FEMS Microbiol Rev. 2001;25:455–501. 10.1016/S0168-6445(01)00063-8. 11524134

[bib187] Walsby AE. Gas vesicles. Microbiol Rev. 1994;58:94–144. 10.1128/mr.58.1.94-144.1994. 8177173 PMC372955

[bib186] Walsby AE. Permeability of gas vesicles to perfluorocyclobutane. Microbiology. 1982;128:1679–84. 10.1099/00221287-128-8-1679.

[bib185] Walsby AE. Structure and function of gas vacuoles. Bacteriol Rev. 1972;36:1–32. 10.1128/br.36.1.1-32.1972. 4337701 PMC378419

[bib183] Walsby AE. The permeability of blue-green algal gas-vacuole membranes to gas. Proc R Soc Lond B Biol Sci. 1969;173:235–55.

[bib184] Walsby AE. The pressure relationships of gas vacuoles. Proc R Soc Lond B Biol Sci. 1971;178:301–26.

[bib188] Wang H, Lindemann E, Liebmann P et al. Methane-cycling microbiomes in soils of the pan-Arctic and their response to permafrost degradation. Commun Earth Environ. 2025;6:748. 10.1038/s43247-025-02765-5. 40969862 PMC12440815

[bib189] Wang Y, Wang Y, Zhou K et al. Isolation of a facultative methanotroph *Methylocystis iwaonis* SD4 from rice rhizosphere and establishment of rapid genetic tools for it. Biotechnol Lett. 2024;46:713–24. 10.1007/s10529-024-03495-y. 38733438

[bib190] Wartiainen I, Hestnes AG, McDonald IR et al. *Methylobacter tundripaludum* sp. nov., a methane-oxidizing bacterium from Arctic wetland soil on the Svalbard islands, Norway (78 N). Int J Syst Evol Microbiol. 2006;56:109–13. 10.1099/ijs.0.63728-0. 16403874

[bib191] Wayne LG, Brenner DJ, Colwell RR et al. Report of the ad hoc committee on reconciliation of approaches to bacterial systematics. Int J Syst Evol Microbiol. 1987;37:463–4. 10.1099/00207713-37-4-463.

[bib192] Weiblen C, Kits KD, Kleiner M et al. Diverse bacteriohemerythrin genes of *Methylomonas denitrificans* FJG1 provide insight into the survival and activity of methanotrophs in low oxygen ecosystems. mBio. 2025:e01533–25. 40996220 10.1128/mbio.01533-25 PMC12607906

[bib193] Whittenbury R, Davies SL, Davey JF. Exospores and cysts formed by methane-utilizing bacteria. J Gen Microbiol. 1970;61:219–26. 10.1099/00221287-61-2-219. 5476892

[bib194] Whittenbury R, Phillips KC, Wilkinson JF. Enrichment, isolation and some properties of methane-utilizing bacteria. J Gen Microbiol. 1970;61:205–18. 10.1099/00221287-61-2-205. 5476891

[bib195] Wood AP, Kelly DP, McDonald IR et al. A novel pink-pigmented facultative methylotroph, *Methylobacterium thiocyanatum* sp. nov., capable of growth on thiocyanate or cyanate as sole nitrogen sources. Arch Microbiol. 1998;169:148–58. 10.1007/s002030050554. 9446686

[bib196] Woodcroft BJ, Singleton CM, Boyd JA et al. Genome-centric view of carbon processing in thawing permafrost. Nature. 2018;560:49–54. 10.1038/s41586-018-0338-1. 30013118

[bib197] Wutkowska M, Daebeler A. Draft genomes of three aerobic methanotrophs from a temperate eutrophic fishpond. Microbiol Resour Announc. 2024;13:e00152–24. 10.1128/mra.00152-24. 38526089 PMC11008168

[bib198] Wutkowska M, Tláskal V, Bordel S et al. Leveraging genome-scale metabolic models to understand aerobic methanotrophs. ISME J. 2024;18:wrae102. 10.1093/ismejo/wrae102. 38861460 PMC11195481

[bib199] Yu Z, Pesesky M, Zhang L et al. A complex interplay between nitric oxide, quorum sensing, and the unique secondary metabolite tundrenone constitutes the hypoxia response in *Methylobacter*. mSystems. 2020;5:e00770–19. 10.1128/msystems.00770-19. 31964770 PMC6977074

[bib200] Zdouc MM, Blin K, Louwen NLL et al. MIBiG 4.0: advancing biosynthetic gene cluster curation through global collaboration. Nucleic Acids Res. 2025;53:D678–90. 10.1093/nar/gkae1115. 39657789 PMC11701617

[bib201] Zheng Y, Huang R, Wang BZ et al. Competitive interactions between methane- and ammonia-oxidizing bacteria modulate carbon and nitrogen cycling in paddy soil. Biogeosciences. 2014;11:3353–68. 10.5194/bg-11-3353-2014.

[bib202] Zheng Y, Wang H, Yu Z et al. Metagenomic insight into environmentally challenged methane-fed microbial communities. Microorganisms. 2020;8:1614. 10.3390/microorganisms8101614. 33092280 PMC7589939

