# Supplementary material for: Uncovering hidden phylo- and ecogenomic diversity of the widespread methanotrophic genus Methylobacter
Source: FEMS Microbiol Ecol. 2025 Dec 13;102(2):fiaf127. doi: 10.1093/femsec/fiaf127 (PMC12810054; doi:10.1093/femsec/fiaf127)
Supplement: fiaf127_Supplemental_Files [file fiaf127_supplemental_files.zip › Supplementary_figures.docx]

**Supplementary figures**

**Supplementary Figure S1** Average amino acid identity (AAI) clustering of the investigated *Methylobacter* spp. genomes and MAGs. The genome AAI similarity matrix was visualised using the Morpheus web-based tool (<https://software.broadinstitute.org/morpheus/>). Hierarchical clustering was performed using one minus Pearson correlation as the distance metric and average linkage. Both rows and columns were clustered to identify coherent genome groups. The name ‘*Methylobacter*’ has been replaced by ‘*M.*’.

**Supplementary Figure S2** Average nucleotide identity (ANIb) clustering of the investigated *Methylobacter* spp. genomes and MAGs. The name ‘*Methylobacter*’ has been replaced by ‘*M.*’.

**Supplementary Figure S3** Phylogenetic trees of genes extracted from the investigated *Methylobacter* spp. **Panel A** shows the full-length 16S rRNA gene (>1,500 bp); **panel B**: subunit A of the particulate methane monooxygenases (pMMO), and **panel C**: subunit A of the sequence-divergent particulate methane monooxygenase (pXMO). The 16S rRNA tree was rooted with *Methylomicrobium lacus* LW14 full 16S rRNA nucleotide sequence (NCBI accession number: NR_042712.1) and indicated by the arrow pointing outwards placed on the first node of the tree. The methane monooxygenase tree was made with all the identified particulate methane monooxygenases, except partial sequences, and was rooted with the AmoA amino acid sequence from *Nitrosomonas europaea* strain ATCC 19718 (UniProtKB/Swiss-Prot: Q04507.2) and indicated by the arrow pointing outwards placed on the first node of the tree. Bootstrap values are represented by circles in three colours: black (>= 95%), grey (80-95%) and white (50-80%). The name ‘*Methylobacter*’ has been replaced by ‘*M.*’.

**
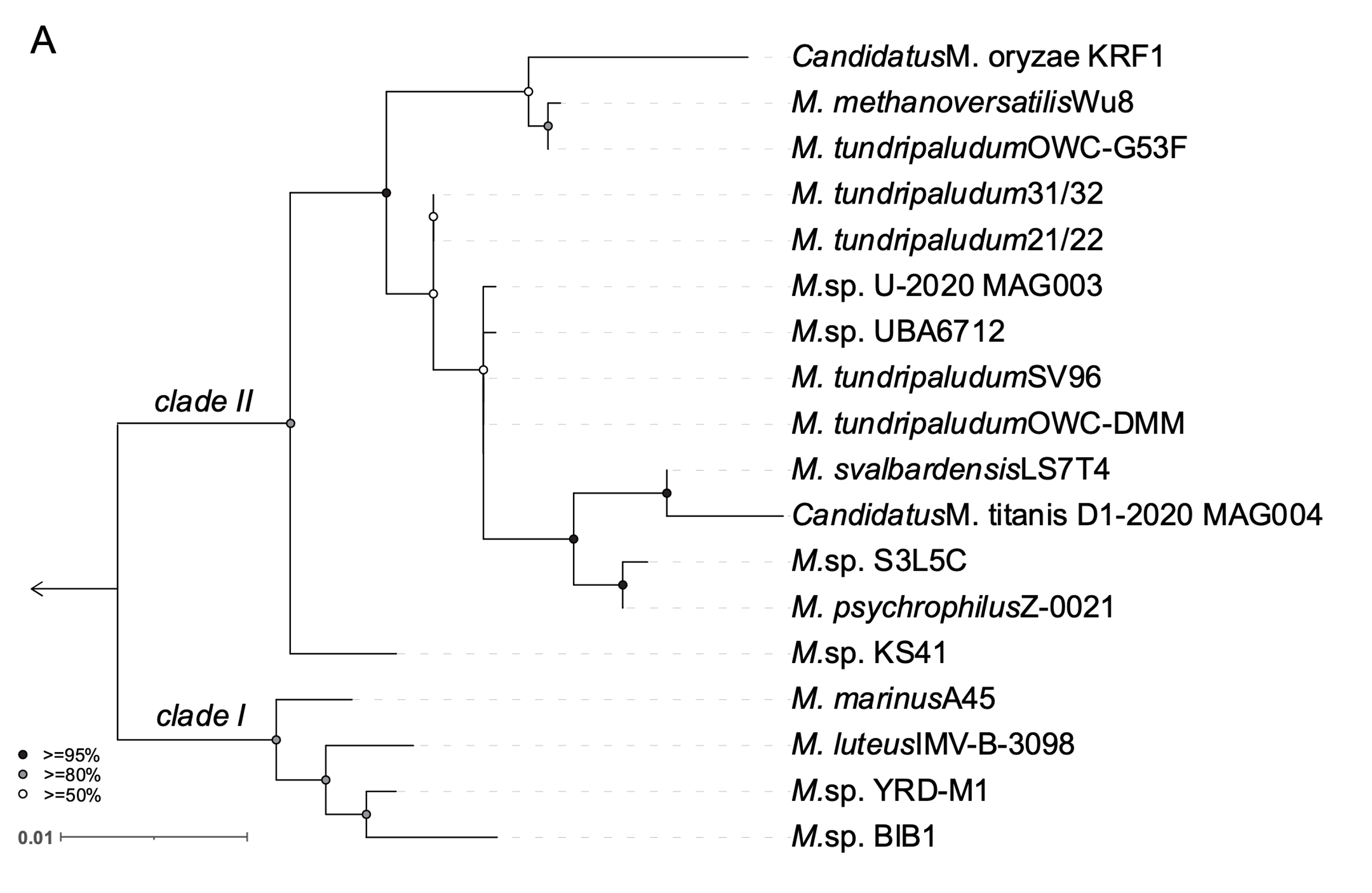
**

**
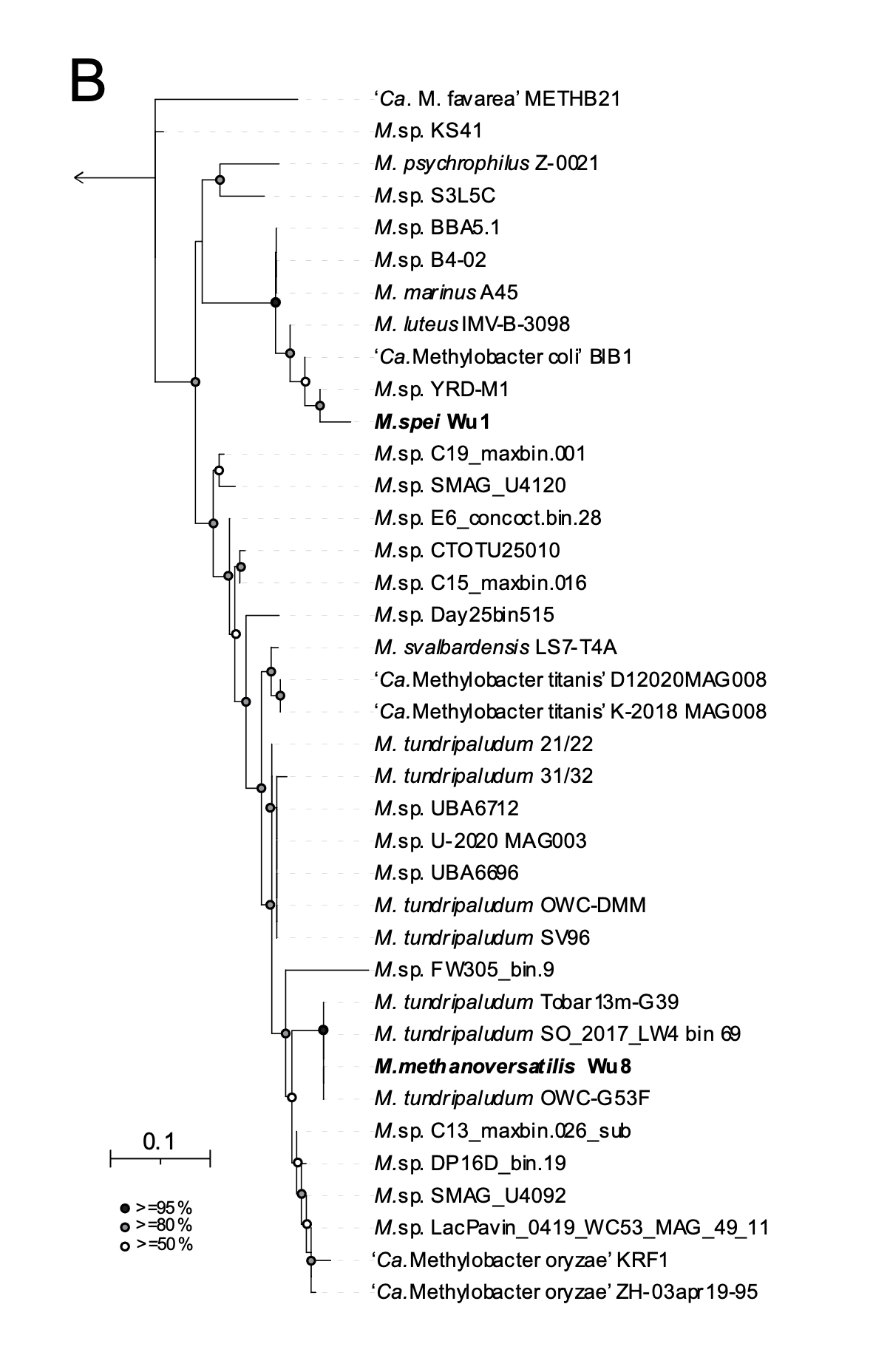
**

**
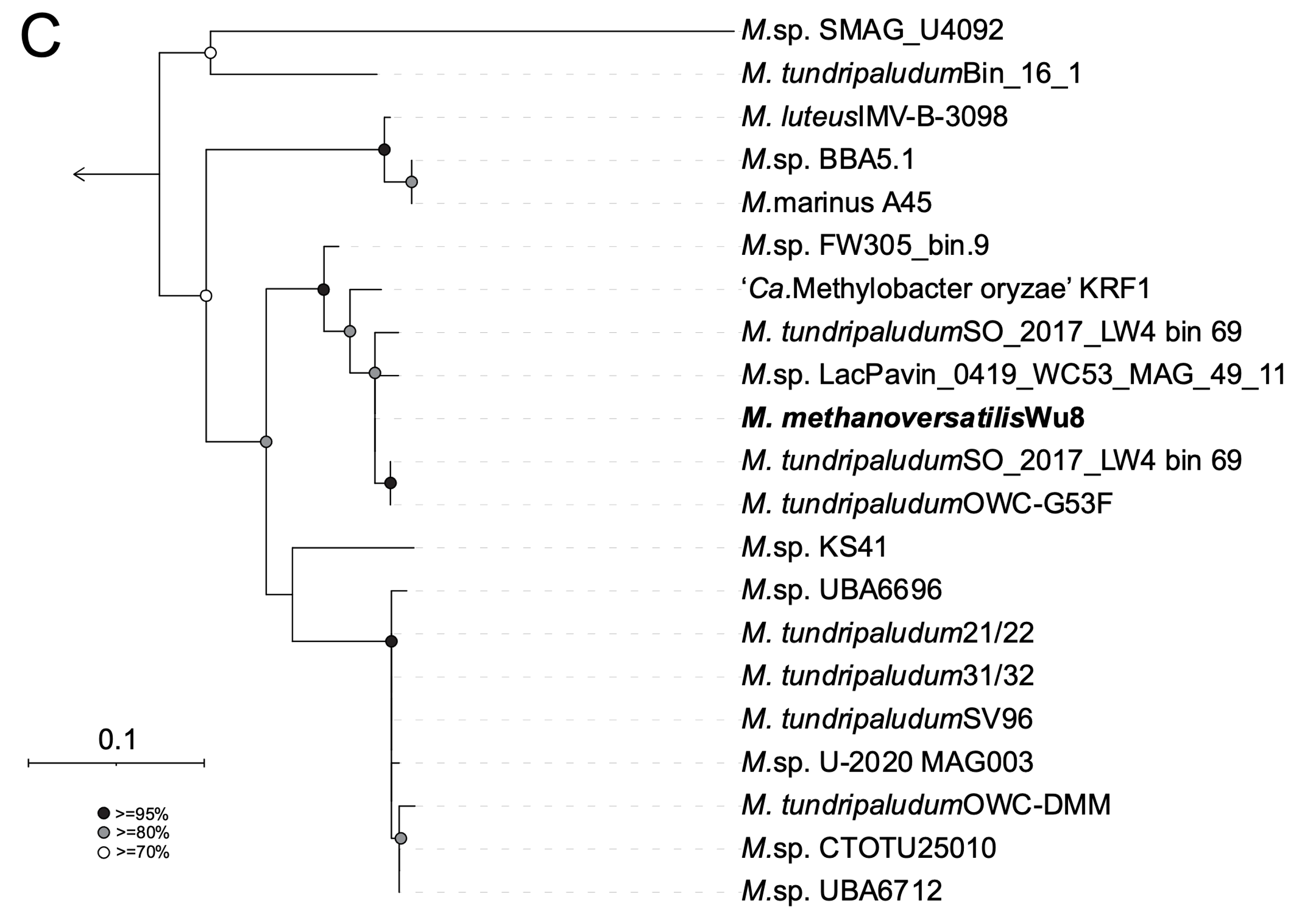
**

**Supplementary Figure S4** Comparison of 16S rRNA genes in *Methylobacter tundripaludum* SV96 and *Methylobacter methanoversatilis* Wu8. Panel A shows multiple sequence alignment of these two sequences with 16S rRNA of *Escherichia coli* JO1859.1 (Brosius *et al.* 1978), highlighting the hypervariable regions V1-V9 in bold (Chakravorty *et al.* 2007). Panel B shows the differences between the two methanotrophic 16S rRNA sequences in red.

1. Multiple sequence alignment of full-length 16S rRNA genes of *Methylobacter tundripaludum* SV96, *Methylobacter methanoversatilis* Wu8, with *Escherichia coli* JO1859.1 (Brosius *et al.*, 1978) highlights the hypervariable regions V1-V9 in **bold** (Chakravorty *et al.*, 2007)

J01859.1 AAATTGAAGAGTTTGATCATGGCTCAGATTGAACGCTGGCGGCAGGCCTAACACATGCAA 60

Wu8 -------AGAGTTTGATCATGGCTCAGATTGAACGCTGGCGGTATGCTTAACACATGCAA 53

SV96 -------AGAGTTTGATCATGGCTCAGATTGAACGCTGGCGGTATGCTTAACACATGCAA 53

*********************************** * ** ************

V1 (69-99)

J01859.1 GTCGAACG**GTAACAGGAAGAAGCTTGCTCTTTGCTGACG**AGTGGCGGACGGGTGAGTAAT 120

Wu8 GTCGAACG**GTAGCAGGCCTTCG-------GGCGCTGACG**AGTGGCGGACGGGTGAGTAAC 106

SV96 GTCGAACG**GTAACAGGCCTTCG-------GGCGCTGACG**AGTGGCGGACGGGTGAGTAAC 106

*********** **** * ***************************

V2 (137-242)

J01859.1 GTCTGGGAAACTGCCT**GATGGAGGGGGATAACTACTGGAAACGGTAGCTAATACCGCATA** 180

Wu8 GCGTAGGAATCTGCCT**CATAGTGGGGGACAACGTGGGGAAACTCACGCTAATACCGCATA** 166

SV96 GCGTAGGAATCTGCCT**CATAGTGGGGGACAACGTGGGGAAACTCACGCTAATACCGCATA** 166

* * **** ****** ** * ****** *** ****** **************

J01859.1 **ACGTCGCAAGACCAAAGAGGGGGACCTTCGGGCCTCTTGCCATCGGATGTGCCCAGATGG** 240

Wu8 **CGCCCTACGGGGGAAAGCGGGGGATCTTCGGACCTCGCGCTATGAGATGAGCCTGCGTTA** 226

SV96 **CGCCCTACGGGGGAAAGCGGGGGATCTTCGGACCTCGCGCTATGAGATGAGCCTGCGTTA** 226

* * **** ****** ****** **** ** ** **** *** *

J01859.1 **GA**TTAGCTAGTAGGTGGGGTAACGGCTCACCTAGGCGACGATCCCTAGCTGGTCTGAGAG 300

Wu8 **GA**TTAGCTAGTTGGTGGGGTAAAGGCCTACCAAGGCGACGATCTATAGCTGGTCTGAGAG 286

SV96 **GA**TTAGCTAGTTGGTAGGGTAAAGGCCTACCAAGGCGACGATCTATAGCTGGTCTGAGAG 286

*********** *** ****** *** *** *********** ***************

J01859.1 GATGACCAGCCACACTGGAACTGAGACACGGTCCAGACTCCTACGGGAGGCAGCAGTGGG 360

Wu8 GACGATCAGCCACACTGGGACTGAGACACGGCCCAGACTCCTACGGGAGGCAGCAGTGGG 346

SV96 GACGATCAGCCACACTGGGACTGAGACACGGCCCAGACTCCTACGGGAGGCAGCAGTGGG 346

** ** ************ ************ ****************************

J01859.1 GAATATTGCACAATGGGCGCAAGCCTGATGCAGCCATGCCGCGTGTATGAAGAAGGCCTT 420

Wu8 GAATATTGGACAATGGGCGAAAGCCTGATCCAGCAATACCGCGTGTGTGAAGAAGGCCTT 406

SV96 GAATATTGGACAATGGGCGAAAGCCTGATCCAGCAATACCGCGTGTGTGAAGAAGGCCTT 406

******** ********** ********* **** ** ******** *************

V3 (433-497)

J01859.1 CGGGTTGTAAAG**TACTTTCAGCGGGGAGGAAGGGAGTAAAGTTAATACCTTTGCTCATTG** 480

Wu8 AGGGTTGTAAAG**CACTTTCAATTGGGAGGAAAACCTACCGGTCAAT-ACCCGGTAGCTTG** 465

SV96 AGGGTTGTAAAG**CACTTTCAATTGGGAGGAAAACCTACCGGTCAAT-ACCCGGTAGCTTG** 465

*********** ******* ******** ** *** * * ***

J01859.1 **ACGTTACCCGCAGAAGA**AGCACCGGCTAACTCCGTGCCAGCAGCCGCGGTAATACGGAGG 540

Wu8 **ACATTACCTTTAGAAGA**AGCACCGGCTAACTCCGTGCCAGCAGCCGCGGTAATACGGAGG 525

SV96 **ACATTACCTTTAGAAGA**AGCACCGGCTAACTCCGTGCCAGCAGCCGCGGTAATACGGAGG 525

** ***** *************************************************

V4 (576-682)

J01859.1 GTGCAAGCGTTAATCGGAATTACTGGGCGTAAAGCG**CACGCAGGCGGTTTGTTAAGTCAG** 600

Wu8 GTGCGAGCGTTAATCGGAATTACTGGGCGTAAAGCG**TGCGTAGGCGGTTCGTTAAGTCAG** 585

SV96 GTGCGAGCGTTAATCGGAATTACTGGGCGTAAAGCG**TGCGTAGGCGGTTCGTTAAGTCAG** 585

**** ******************************* ** ******** **********

J01859.1 **ATGTGAAATCCCCGGGCTCAACCTGGGAACTGCATCTGATACTGGCAAGCTTGAGTCTCG** 660

Wu8 **ATGTGAAAGCCCTGGGCTCAACCTGGGAACGGCATTTGAAACTGGCGGACTAGAGTTTAG** 645

SV96 **ATGTGAAAGCCCTGGGCTCAACCTGGGAACTGCATTTGAAACTGGCGGACTAGAGTTTAG** 645

******** *** ***************** **** *** ****** ** **** * *

J01859.1 **TAGAGGGGGGTAGAATTCCAGG**TGTAGCGGTGAAATGCGTAGAGATCTGGAGGAATACCG 720

Wu8 **TAGAGGGGAGTGGAATTTCAGG**TGTAGCGGTGAAATGCGTAGATATCTGAAGGAACACCA 705

SV96 **TAGAGGGGAGTGGAATTTCAGG**TGTAGCGGTGAAATGCGTAGATATCTGAAGGAACACCA 705

******** ** ***** ************************* ***** ***** ***

J01859.1 GTGGCGAAGGCGGCCCCCTGGACGAAGACTGACGCTCAGGTGCGAAAGCGTGGGGAGCAA 780

Wu8 GTGGCGAAGGCGACTCCCTGGACTAGAACTGACGCTGAGGTACGAAAGCGTGGGTAGCAA 765

SV96 GTGGCGAAGGCGACTCCCTGGACTAGAACTGACGCTGAGGTACGAAAGCGTGGGTAGCAA 765

************ * ******** * ********* **** ************ *****

V5 (822-879)

J01859.1 ACAGGATTAGATACCCTGGTAGTCCACGCCGTAAACGATGTC**GACTTGGAGGTTGTGCCC** 840

Wu8 ACAGGATTAGATACCCTGGTAGTCCACGCCGTAAACGATGTC**AACTAGCCGTTGGTCCTA** 825

SV96 ACAGGATTAGATACCCTGGTAGTCCACGCCGTAAACGATGTC**GACTAGCCGTTGGTCCTA** 825

****************************************** *** * * * ** *

J01859.1 **TTGAG-GCGTGGCTTCCGGAGCTAACGCGTTAAGTCGACC**GCCTGGGGAGTACGGCCGCA 899

Wu8 **TTTACAGGATTAGTGGCGCAGCTAACGCATTAAGTTGACC**GCCTGGGGAGTACGGCCGCA 885

SV96 **TTTACAGGATTAGTGGCGCAGCTAACGCATTAAGTCGACC**GCCTGGGGAGTACGGCCGCA 885

** * * * * ** ********* ****** ************************

J01859.1 AGGTTAAAACTCAAATGAATTGACGGGGGCCCGCACAAGCGGTGGAGCATGTGGTTTAAT 959

Wu8 AGGTTAAAACTCAAATGAATTGACGGGGGCCCGCACAAGCGGTGGAGCATGTGGTTTAAT 945

SV96 AGGTTAAAACTCAAATGAATTGACGGGGGCCCGCACAAGCGGTGGAGCATGTGGTTTAAT 945

************************************************************

V6 (986-1043)

J01859.1 TCGATGCAACGCGAAGAACCTTACCT**GGTCTTGACATCCACGGAAGTTTTCAGAGATGAG** 1019

Wu8 TCGATGCAACGCGAAGAACCTTACCT**ACCCTTGACATCCAGAGAATCTGTTAGAGATAGT** 1005

SV96 TCGATGCAACGCGAAGAACCTTACCT**ACCCTTGACATCCAGAGAATCTGTTAGAGATAGT** 1005

************************** *********** *** * * ******

J01859.1 **AATGTGCCTTCGGGAACCGTGAG**ACAGGTGCTGCATGGCTGTCGTCAGCTCGTGTTGTGA 1079

Wu8 **AGAGTGCCTTCGGGAACTCTGAG**ACAGGTGCTGCATGGCTGTCGTCAGCTCGTGTCGTGA 1065

SV96 **AGAGTGCCTTCGGGAGCTCTGAG**ACAGGTGCTGCATGGCTGTCGTCAGCTCGTGTCGTGA 1065

* ************ * ************************************ ****

V7 (1117-1173)

J01859.1 AATGTTGGGTTAAGTCCCGCAACGAGCGCAACCCTTA**TCCTTTGTTGCCAGCGGTC-CGG** 1138

Wu8 GATGTTGGGTTAAGTCCCGTAACGAGCGCAACCCTTA**TCCTTAGTTGCCAGCACATTATG** 1125

SV96 GATGTTGGGTTAAGTCCCGTAACGAGCGCAACCCTTA**TCCTTAGTTGCCAGCGGGTAATG** 1125

****************** ********************** ********* *

J01859.1 **CCGGGAACTCAAAGGAGACTGCCAGTGATAAACTG**GAGGAAGGTGGGGATGACGTCAAGT 1198

Wu8 **GTGGGAACTCTAGGGAGACTGCCGGTGATAAACCG**GAGGAAGGTGGGGACGACGTCAAGT 1185

SV96 **CCGGGAACTCTAGGGAGACTGCCGGTGATAAACCG**GAGGAAGGTGGGGACGACGTCAAGT 1185

******** * ********** ********* *************** **********

V8 (1243-1294)

J01859.1 CATCATGGCCCTTACGACCAGGGCTACACACGTGCTACAATGGC**GCATACAAAGAGAAGC** 1258

Wu8 CATCATGGCCCTTATGGGTAGGGCTACACACGTGCTACAATGGT**CGGTACAGAGGGCTGC** 1245

SV96 CATCATGGCCCTTATGGGTAGGGCTACACACGTGCTACAATGGC**CGGTACAGAGGGCTGC** 1245

************** * ************************ **** ** * **

J01859.1 **GACCTCGCGAGAGCAAGCGGACCTCATAAAGTGCGT**CGTAGTCCGGATTGGAGTCTGCAA 1318

Wu8 **GAAACCGCGAGGTCAAGCAAATCCCAGAAAGCCGAT**CCTAGTCCGGATTGGAGTCTGCAA 1305

SV96 **GAACTTGCGAAAGTAAGCAAATCCCAGAAAGCCGGT**CCTAGTCCGGATTGGAGTCTGCAA 1305

** **** **** * * ** **** ** **********************

J01859.1 CTCGACTCCATGAAGTCGGAATCGCTAGTAATCGTGGATCAGAATGCCACGGTGAATACG 1378

Wu8 CTCGACTCCATGAAGTCGGAATCGCTAGTAATCGCGAATCAGAATGTCGCGGTGAATACG 1365

SV96 CTCGACTCCATGAAGTCGGAATCGCTAGTAATCGCGAATCAGAATGTCGCGGTGAATACG 1365

********************************** * ********* * ***********

V9 (1435-1465)

J01859.1 TTCCCGGGCCTTGTACACACCGCCCGTCACACCATGGGAGTGGGTTGCAAAAGAAG**TAGG** 1438

Wu8 TTCCCGGGCCTTGTACACACCGCCCGTCACACCATGGGAGTGGGTTGCAAAAGAAG**TGGG** 1425

SV96 TTCCCGGGCCTTGTACACACCGCCCGTCACACCATGGGAGTGGGTTGCAAAAGAAG**TGGG** 1425

********************************************************* **

J01859.1 **TAGCTTAACCTTCGGGAGGGCGCTTAC**CACTTTGTGATTCATGACTGGGGTGAAGTCGTA 1498

Wu8 **TAGTCTAACCTTCGGGAGGGCGCTCAC**CACTTTGTGATTCATGACTGGGGTGAAGTCGTA 1485

SV96 **TAGTCTAACCTTCGGGAGGGCGCTCAC**CACTTTGTGATTCATGACTGGGGTGAAGTCGTA 1485

*** ******************* ***********************************

J01859.1 ACAAGGTAACCGTAGGGGAACCTGCGGTTGGATCACCTCCTTA 1541

Wu8 ACAAGGTAGCCCTAGGGGAACCTGGGGCTGGATCACCT----- 1523

SV96 ACAAGGTAGCCCTAGGGGAACCTGGGGCTGGATCACCT----- 1523

******** ** ************ ** **********

1. The nucleotide differences in full-length 16S rRNA genes of *Methylobacter tundripaludum* SV96 and *Methylobacter methanoversatilis* Wu8 (marked in red)

SV96 AGAGTTTGATCATGGCTCAGATTGAACGCTGGCGGTATGCTTAACACATGCAAGTCGAAC 60

Wu8 AGAGTTTGATCATGGCTCAGATTGAACGCTGGCGGTATGCTTAACACATGCAAGTCGAAC 60

************************************************************

V1

SV96 G**GTAACAGGCCTTCGGGCGCTGACG**AGTGGCGGACGGGTGAGTAACGCGTAGGAATCTGC 120

Wu8 G**GTAGCAGGCCTTCGGGCGCTGACG**AGTGGCGGACGGGTGAGTAACGCGTAGGAATCTGC 120

****!*******************************************************

V2

SV96 CT**CATAGTGGGGGACAACGTGGGGAAACTCACGCTAATACCGCATACGCCCTACGGGGGA** 180

Wu8 CT**CATAGTGGGGGACAACGTGGGGAAACTCACGCTAATACCGCATACGCCCTACGGGGGA** 180

************************************************************

SV96 **AAGCGGGGGATCTTCGGACCTCGCGCTATGAGATGAGCCTGCGTTAGA**TTAGCTAGTTGG 240

Wu8 **AAGCGGGGGATCTTCGGACCTCGCGCTATGAGATGAGCCTGCGTTAGA**TTAGCTAGTTGG 240

************************************************************

SV96 TAGGGTAAAGGCCTACCAAGGCGACGATCTATAGCTGGTCTGAGAGGACGATCAGCCACA 300

Wu8 TGGGGTAAAGGCCTACCAAGGCGACGATCTATAGCTGGTCTGAGAGGACGATCAGCCACA 300

*!**********************************************************

SV96 CTGGGACTGAGACACGGCCCAGACTCCTACGGGAGGCAGCAGTGGGGAATATTGGACAAT 360

Wu8 CTGGGACTGAGACACGGCCCAGACTCCTACGGGAGGCAGCAGTGGGGAATATTGGACAAT 360

************************************************************

V3

SV96 GGGCGAAAGCCTGATCCAGCAATACCGCGTGTGTGAAGAAGGCCTTAGGGTTGTAAAG**CA** 420

Wu8 GGGCGAAAGCCTGATCCAGCAATACCGCGTGTGTGAAGAAGGCCTTAGGGTTGTAAAG**CA** 420

************************************************************

SV96 **CTTTCAATTGGGAGGAAAACCTACCGGTCAATACCCGGTAGCTTGACATTACCTTTAGAA** 480

Wu8 **CTTTCAATTGGGAGGAAAACCTACCGGTCAATACCCGGTAGCTTGACATTACCTTTAGAA** 480

************************************************************

SV96 **GA**AGCACCGGCTAACTCCGTGCCAGCAGCCGCGGTAATACGGAGGGTGCGAGCGTTAATC 540

Wu8 **GA**AGCACCGGCTAACTCCGTGCCAGCAGCCGCGGTAATACGGAGGGTGCGAGCGTTAATC 540

************************************************************

V4

SV96 GGAATTACTGGGCGTAAAGCG**TGCGTAGGCGGTTCGTTAAGTCAGATGTGAAAGCCCTGG** 600

Wu8 GGAATTACTGGGCGTAAAGCG**TGCGTAGGCGGTTCGTTAAGTCAGATGTGAAAGCCCTGG** 600

************************************************************

SV96 **GCTCAACCTGGGAACTGCATTTGAAACTGGCGGACTAGAGTTTAGTAGAGGGGAGTGGAA** 660

Wu8 **GCTCAACCTGGGAACGGCATTTGAAACTGGCGGACTAGAGTTTAGTAGAGGGGAGTGGAA** 660

***************!********************************************

SV96 **TTTCAGG**TGTAGCGGTGAAATGCGTAGATATCTGAAGGAACACCAGTGGCGAAGGCGACT 720

Wu8 **TTTCAGG**TGTAGCGGTGAAATGCGTAGATATCTGAAGGAACACCAGTGGCGAAGGCGACT 720

************************************************************

SV96 CCCTGGACTAGAACTGACGCTGAGGTACGAAAGCGTGGGTAGCAAACAGGATTAGATACC 780

Wu8 CCCTGGACTAGAACTGACGCTGAGGTACGAAAGCGTGGGTAGCAAACAGGATTAGATACC 780

************************************************************

V5

SV96 CTGGTAGTCCACGCCGTAAACGATGTC**GACTAGCCGTTGGTCCTATTTACAGGATTAGTG** 840

Wu8 CTGGTAGTCCACGCCGTAAACGATGTC**AACTAGCCGTTGGTCCTATTTACAGGATTAGTG** 840

***************************!********************************

SV96 **GCGCAGCTAACGCATTAAGTCGACC**GCCTGGGGAGTACGGCCGCAAGGTTAAAACTCAAA 900

Wu8 **GCGCAGCTAACGCATTAAGTTGACC**GCCTGGGGAGTACGGCCGCAAGGTTAAAACTCAAA 900

******************** ***************************************

SV96 TGAATTGACGGGGGCCCGCACAAGCGGTGGAGCATGTGGTTTAATTCGATGCAACGCGAA 960

Wu8 TGAATTGACGGGGGCCCGCACAAGCGGTGGAGCATGTGGTTTAATTCGATGCAACGCGAA 960

************************************************************

V6

SV96 GAACCTTACCT**ACCCTTGACATCCAGAGAATCTGTTAGAGATAGTAGAGTGCCTTCGGGA** 1020

Wu8 GAACCTTACCT**ACCCTTGACATCCAGAGAATCTGTTAGAGATAGTAGAGTGCCTTCGGGA** 1020

************************************************************

SV96 **GCTCTGAG**ACAGGTGCTGCATGGCTGTCGTCAGCTCGTGTCGTGAGATGTTGGGTTAAGT 1080

Wu8 **ACTCTGAG**ACAGGTGCTGCATGGCTGTCGTCAGCTCGTGTCGTGAGATGTTGGGTTAAGT 1080

!***********************************************************

V7

SV96 CCCGTAACGAGCGCAACCCTTA**TCCTTAGTTGCCAGCGGGTAATGCCGGGAACTCTAGGG** 1140

Wu8 CCCGTAACGAGCGCAACCCTTA**TCCTTAGTTGCCAGCACATTATGGTGGGAACTCTAGGG** 1140

*************************************!!!*!***!!*************

SV96 **AGACTGCCGGTGATAAACCG**GAGGAAGGTGGGGACGACGTCAAGTCATCATGGCCCTTAT 1200

Wu8 **AGACTGCCGGTGATAAACCG**GAGGAAGGTGGGGACGACGTCAAGTCATCATGGCCCTTAT 1200

************************************************************

V8

SV96 GGGTAGGGCTACACACGTGCTACAATGGC**CGGTACAGAGGGCTGCGAACTTGCGAAAGTA** 1260

Wu8 GGGTAGGGCTACACACGTGCTACAATGGT**CGGTACAGAGGGCTGCGAAACCGCGAGGTCA** 1260

**************************** *******************!!!****!!!!*

SV96 **AGCAAATCCCAGAAAGCCGGT**CCTAGTCCGGATTGGAGTCTGCAACTCGACTCCATGAAG 1320

Wu8 **AGCAAATCCCAGAAAGCCGAT**CCTAGTCCGGATTGGAGTCTGCAACTCGACTCCATGAAG 1320

*******************!****************************************

SV96 TCGGAATCGCTAGTAATCGCGAATCAGAATGTCGCGGTGAATACGTTCCCGGGCCTTGTA 1380

Wu8 TCGGAATCGCTAGTAATCGCGAATCAGAATGTCGCGGTGAATACGTTCCCGGGCCTTGTA 1380

************************************************************

V9

SV96 CACACCGCCCGTCACACCATGGGAGTGGGTTGCAAAAGAAG**TGGGTAGTCTAACCTTCGG** 1440

Wu8 CACACCGCCCGTCACACCATGGGAGTGGGTTGCAAAAGAAG**TGGGTAGTCTAACCTTCGG** 1440

************************************************************

SV96 **GAGGGCGCTCAC**CACTTTGTGATTCATGACTGGGGTGAAGTCGTAACAAGGTAGCCCTAG 1500

Wu8 **GAGGGCGCTCAC**CACTTTGTGATTCATGACTGGGGTGAAGTCGTAACAAGGTAGCCCTAG 1500

************************************************************

SV96 GGGAACCTGGGGCTGGATCACCT 1523

Wu8 GGGAACCTGGGGCTGGATCACCT 1523

***********************

**Supplementary Figure S5** Unrooted phylogenetic trees of ATP synthase subunit 3 identified in *Methylobacter* spp. Panel A shows identified H^+^-ATP synthase AtpE. Panel B shows identified Na^+^-ATP synthase AtpE. Verified H^+^-ATP synthase AtpE sequence from *Escherichia coli* K12 (P68699, ATPL_ECOLI) was included into the tree in panel A. Panel B includes several sequences verified as Na^+^-ATP synthase AtpE sequences from *Acetobacterium woodie* strain ATCC 29683 (H6LFT2, H6LFT2_ACEWD), *Natranaerobius thermophilus* strain ATCC BAA-1301 (B2A3G7 (ATPL_NATTJ)), *Propionigenium modestum* strain ATCC 35614 (P21905, ATPL_PROMO), and *Ilyobacter tartaricus* strain ATCC 35898 (Q8KRV3, ATPL_ILYTA). The scale bar indicated 0.1 amino acid substitutions per site. Branch bootstrap support is indicated on the nodes as black (≥95%), grey (≥80%), or white (>50%) circles. The name ‘*Methylobacter*’ has been replaced by ‘*M.*’.

**
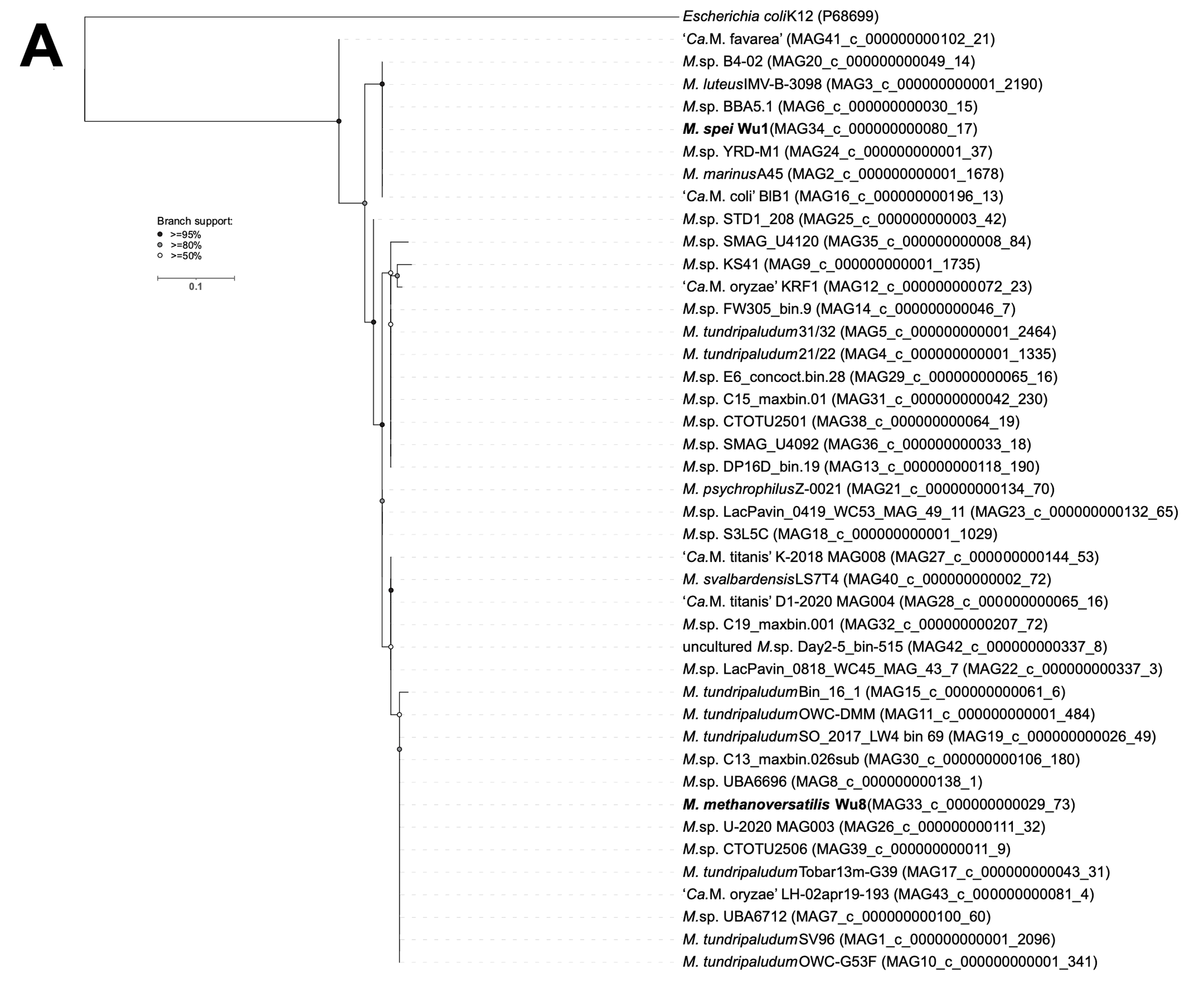

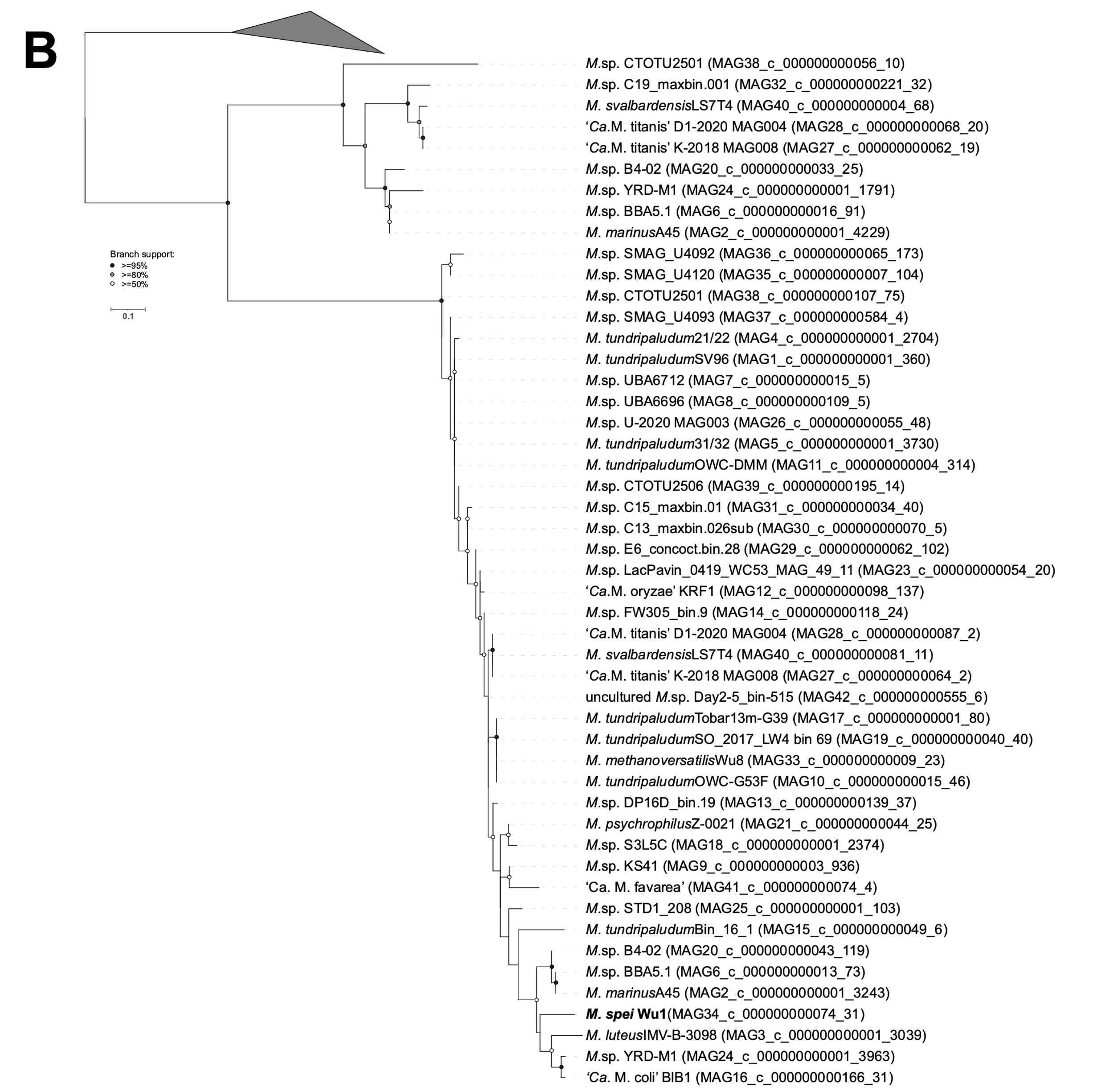
**

**Supplementary Figure S6** Phylogenetic tree of thiosulphohydrolase (SoxB, also known as S-sulphosulphanyl-L-cysteine sulphohydrolase, EC: 3.12.1.1). SoxB sequence from *Thiobacillus denitrificans* (WP_018077747.1) was one of the sequences used in the curated soxB sequence collection. As an outgroup, we used a verified sequence of periplasmic bacterial 5'-nucleotidase (Q9XZ43), also known as UDP-sugar hydrolase, that belongs to a superior protein family 5'-Nucleotidase/apyrase (IPR006179), which includes thiosulfohydrolase SoxB (IPR030998). The outgroup is indicated by the arrow pointing outwards on the first node of the tree. The scale bar indicated 1 amino acid substitution per site. Branch bootstrap support is indicated on the nodes as black (≥95%), grey (≥80%), or white (>50%) circles. The name ‘*Methylobacter*’ has been replaced by ‘*M.*’.

**
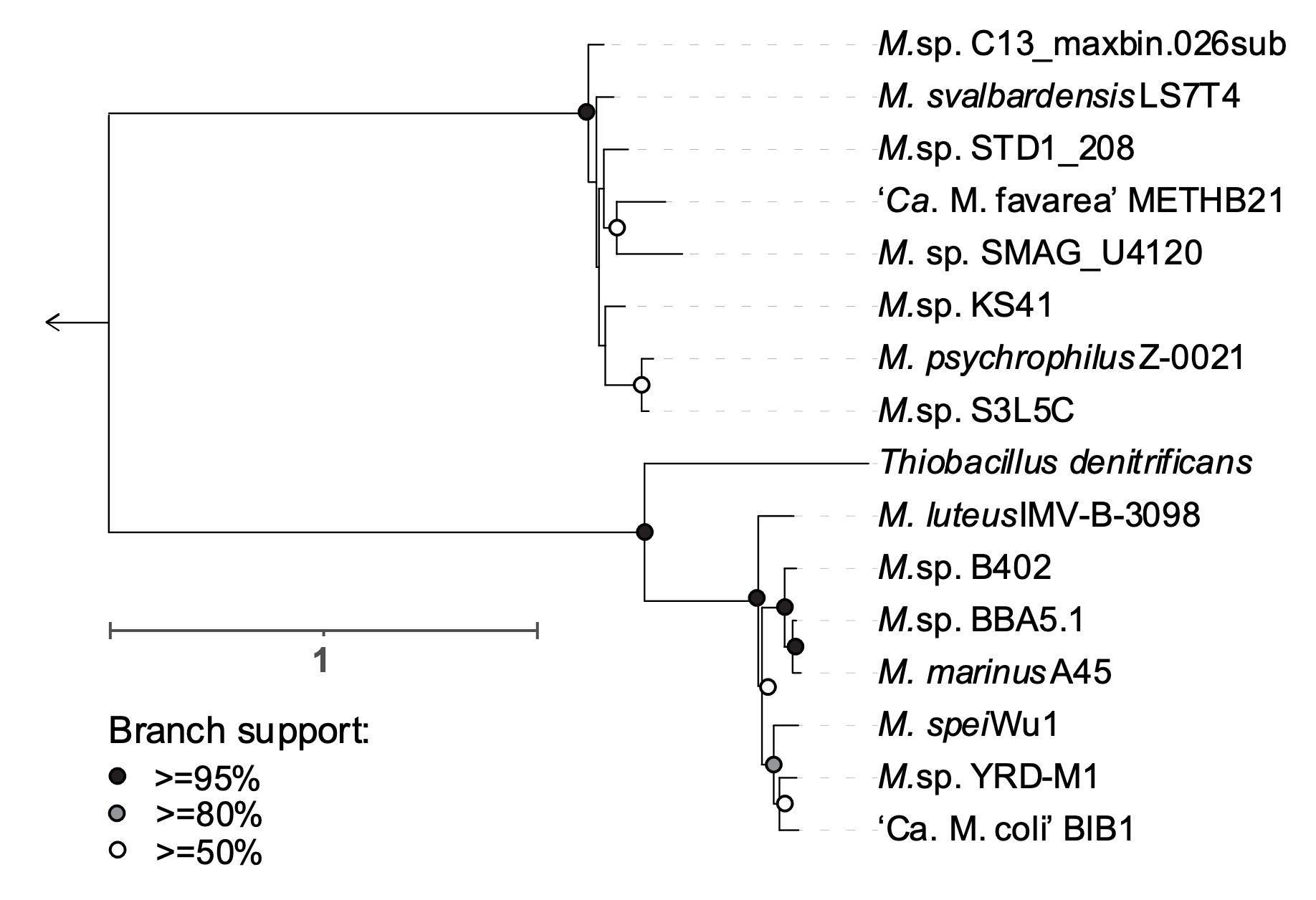
**

**Supplementary Figure S7** Genome-based predictions of putative optimal temperature (A), pH (B) and salinity (C) values for culturing the investigated *Methylobacter* spp. Black dots indicate genome-based *in silico* predicted optimal growth values for each parameter. Green lines indicate experimentally verified growth ranges of cultured species, green dot indicates optimal growth conditions, and empty circles indicate validated inhibitory concentrations. The name ‘*Methylobacter*’ has been replaced by ‘*M.*’.

References:

Brosius J, Palmer ML, Kennedy PJ et al. Complete nucleotide sequence of a 16S ribosomal RNA gene from *Escherichia coli*. Proc Natl Acad Sci USA 1978;75:4801-5. <https://doi.org/10.1073/pnas.75.10.4801>.

Chakravorty S, Helb D, Burday M et al. A detailed analysis of 16S ribosomal RNA gene segments for the diagnosis of pathogenic bacteria. J Microbiol Methods 2007;69:330-9. <https://doi.org/10.1016/j.mimet.2007.02.005>.
